# Informing the measurement of wellbeing among young people living with HIV in sub-Saharan Africa for policy evaluations: a mixed-methods systematic review

**DOI:** 10.1186/s12955-020-01352-w

**Published:** 2020-05-05

**Authors:** Darshini Govindasamy, Janet Seeley, Ioana D. Olaru, Alison Wiyeh, Catherine Mathews, Giulia Ferrari

**Affiliations:** 1 Department of Global Health and Development, Faculty of Public Health and Policy, London School of Hygiene and Tropical Medicine, London, UK; 2 Health Systems Research Unit, South African Medical Research Council, Francie van Zijl Drive, Parow Valley, PO Box 19070, Tygerberg, 7501 South Africa; 3 Adolescent Health Research Unit, Department of Child and Adolescent Psychiatry, University of Cape Town, Cape Town, South Africa; 4 Department of Clinical Research, Faculty of Infectious and Tropical Diseases, London School of Hygiene and Tropical Medicine, London, UK; 5 Biomedical Research and Training Institute, Harare, Zimbabwe; 6 Cochrane South Africa, South African Medical Research Council, Tygerberg, South Africa; 7 Department of Epidemiology, University of Washington School of Public Health, Seattle, Washington USA

**Keywords:** Wellbeing, Mental health, Dimensions, Measurement, Young people living with HIV, Mixed-methods review, Sub-Saharan Africa

## Abstract

Young people living with HIV (YPLHIV) in sub-Saharan Africa (SSA) are at high risk of having a poor quality of life. Addressing wellbeing explicitly within HIV/AIDS policies could assist mitigation efforts. However, guidance on wellbeing measures to evaluate policies for YPLHIV is scarce. The aims of this mixed-methods review were to identify: i) key dimensions of wellbeing and ii) wellbeing measures that align to these dimensions among YPLHIV (15–24 years) in SSA. We searched six social science and medical databases, including grey literature. We included studies that examined correlates and lived experiences of wellbeing, among YPLHIV in SSA, from January 2000 to May 2019. Two reviewers independently screened abstracts and full texts and assessed methodological quality of included articles. We analysed quantitative and qualitative data using descriptive and meta-ethnographic approaches, respectively. Thereafter, we integrated findings using a framework approach. We identified 6527 citations. Of these, 10 quantitative and 30 qualitative studies were included. Being male, higher educational status, less stigma and more social support were likely correlates of wellbeing. Themes that shaped experiences suggestive of wellbeing were: 1) acceptance and belonging— stigma, social support; 2) coping; 3) standard of living. Our final synthesis found that the following dimensions potentially characterise wellbeing: self-acceptance, belonging, autonomy; positive relations, environmental mastery, purpose in life. Wellbeing for YPLHIV is multi-dimensional and relational. Relevant measures include the Personal Wellbeing Index, Ryff’s Psychological Wellbeing Scale and Mental Health Continuum Short Form. However, psychometric evaluations of these scales among YPLHIV in SSA are needed.

## Introduction

Adolescence and young adulthood are critical periods for wellbeing, an indicator of the quality of life. This life stage is important for building human and social capital, resources that can sustain wellbeing in adulthood [1–3]. For young people living with HIV (YPLHIV), who because of greater access to anti-retroviral therapy (ART), can expect to reach adulthood, building and sustaining wellbeing as they grow older is crucial. In sub-Saharan Africa (SSA) there are an estimated 2.8 million YPLHIV aged 15–24 years [4]. Many YPLHIV in SSA are at high risk for HIV-related mortality, morbidity [5] and poor quality of life due to the sub-optimal access to treatment and psycho-social services, including exposure to multiple stigmas [6–9]. According to the United Nations [10], the number of YPLHIV in Africa is projected to increase by 44% between 2015 and 2030. Models predict that investment in the quality of life among the growing youth population in SSA, particularly via addressing health needs, could increase labour productivity and resultantly accelerate economic growth [11]. If long-run economic growth is to be achieved, then public health policies need to also promote the wellbeing among YPLHIV, a vulnerable and growing population in this region.

In social psychology, wellbeing denotes a sense of thriving in multiple life domains such as family, career and health [12]. Two constructs of wellbeing exist, subjective wellbeing (SWB) which focuses on people’s emotional and cognitive evaluations of their lives [13] and psychological wellbeing (PWB) which emphasise positive functioning, relationships and human potential [14, 15]. The wellbeing of YPLHIV in SSA is likely to be compromised given the impact HIV/AIDS has on key dimensions of wellbeing such as finding meaning, self-acceptance and maintenance of positive caregiver and peer relationships [16]. Importantly, emerging empirical evidence has shown that YPLHIV in this setting are at high risk for depression and anxiety [17–19] and suicidal ideation [20]. Negative mental health functioning is one of the strongest predictors of wellbeing [21], and if experienced during adolescence may lead to reduced wellbeing in adulthood [2, 22], and subsequently unfavourable labour-market outcomes such as low earnings [23–25]. The risk of lowered wellbeing among YPLHIV is of major concern as econometric evidence has shown that the stocks and intergenerational transfer of human capital to young people in this region has already been eroded by the HIV/AIDS epidemic [26, 27].

In line with Sustainable Development Goal (SDG) 3 (“Ensure healthy lives and promote well-being for all at all ages”), wellbeing has emerged as a major policy outcome [28, 29]. Substantial investment and progress has been made in reducing the HIV epidemic among young people (SDG 3.3) via scale-up of HIV testing and access to treatment [30, 31]. Yet, promoting mental health and wellbeing (SDG 3.4) among young people has received very little attention. There is growing recognition for the need to include quality of life as an HIV/AIDS programme target [32, 33]. Current multi-sectoral HIV programmes are focused on reducing new HIV-infections among adolescent girls and young women via improving access to health and social services, including economic opportunities [34]. However, monitoring and evaluation frameworks of these programmes do not explicitly include wellbeing as an outcome, a recommended measure in the field of economics for measuring social progress [35]. Valuing only narrow health outcomes in health economic analyses may fail to capture the broader impacts of the multi-sectoral initiatives that go beyond health [36, 37]. From a public health financing perspective, understanding the impact of HIV policies on wellbeing could provide a range of ways to direct and strengthen investments for YPLHIV.

Wellbeing measures are used in economic policy evaluations as empirical approximations of individual welfare (i.e. the satisfaction (utility) gained from consuming a good or service) [38]. Of the few health-related economic studies that have examined wellbeing among children and adolescents, most have used uni-dimensional life satisfaction measures [39, 40]. However, several multi-dimensional wellbeing measures developed for young people in the Global North exist in the field of social psychology, these include multi-dimensional SWB scales (i.e. Student Life Satisfaction Scale [41], Personal Wellbeing Index [42]), and PWB measures (Ryff Scale of PWB [43], Mental Health Continuum Short-Form [44]). In the field of HIV/AIDS epidemiology, health-related quality of life measures that encompass some wellbeing dimensions such as emotional and social functioning have been recommended [45] and applied among adolescents living with HIV, these include the Paediatric Quality of Life Inventory [46], KIDSCREEN [47], WHO Quality of Life [48]. However, there is a lack of guidance on which wellbeing measures, underpinned by wellbeing theory, are appropriate for health economic surveys seeking to evaluate the impact of HIV policies on the wellbeing of YPLHIV.

Wellbeing may be conceptualised, experienced, and valued differently across cultures. A better understanding of the correlates and local experiences of wellbeing among YPLHIV in SSA and how these relate to international conceptualisations of wellbeing is required in the selection of culturally sensitive, yet generalisable, measures of wellbeing for SSA. While several studies have explored wellbeing among young people in this sub-region [49, 50], to the best of our knowledge, no study has systematically reviewed the evidence on what constitutes wellbeing among YPLHIV on the continent. The objectives of this mixed-methods review are to identify and critically assess: 1) quantitative evidence on the correlates of wellbeing among YPLHIV; 2) qualitative evidence on the lived experiences of wellbeing among YPLHIV; 3) integrate these two strands of evidence and identify appropriate measures of wellbeing based on key dimensions of wellbeing relevant to YPLHIV.

The main reason for using quantitative and qualitative evidence in this review is to provide a broad perspective in order to gain a detailed and thorough understanding of the indicators of wellbeing and potential mechanisms by which factors influence wellbeing [51–53]. The findings from this review could guide the selection of wellbeing measures for policy evaluations focused on YPLHIV.

## Methods

The quantitative review was prepared in accordance with Preferred Reporting Items for Systematic Reviews and Meta-analysis (PRISMA) checklist [54] (Additional file 1). Whereas the qualitative review adhered to the Enhancing transparency in reporting the synthesis of qualitative research (ENTREQ) statement [55] (Additional file 2). Reporting of this review was informed by mixed-method review guidelines [56].

### Epistemological perspective and study design

We adopted a relational wellbeing perspective as increasing research from the fields of social psychology, anthropology and international development have highlighted that relationships are central to the experiences of wellbeing in low- and middle-income countries, particularly studies from SSA [57–59]. We drew on the relationality meaning model as proposed by Wissing [60] as relationships and connections have been shown to be central to formation of wellbeing among young people [61–65]. According to the relationality meaning model, relationships, with self (intrapersonal) and others (interpersonal) are at the centre of a meaningful life, and play an important role in the connections between people and context (i.e. social, cultural, ecological, physical, spiritual) [60]. This model has a strong PWB orientation and emphasises three key elements: 1. *meaning of life* (experienced in the connectedness to something larger than life and the realisation of values); 2. *meaning in life* (experienced in the belongingness and relatedness to other people); and 3. *meaning to life* (expressed via actions such as expressions of affection, longing for belongingness, building relationships). This model hypothesises that meaning of life facilitates the construction of meaning in life which in turn directs activities to provide meaning to life [60]. We specifically applied elements of this model to understand the pathways to wellbeing in order to elucidate dimensions that are relevant in capturing the wellbeing among YPLHIV.

The mixed-methods approach used was a segregated (convergent) design [66]. We selected this design because we regarded quantitative and qualitative research as complementary. However, we conducted the quantitative and qualitative review separately as different approaches were used and the review criteria differed. For the quantitative review, we followed systematic review methods used in evidenced-based medicine [67]. Whereas for the qualitative review, we used a flexible meta-ethnography design [68]. Quantitative and qualitative studies were retrieved, analysed and synthesised separately, prior to the final synthesis [66].

### Eligibility

The criteria for inclusion of studies are summarised in Table 1.

**Table 1:** Eligibility criteria for quantitative and qualitative studies

	Inclusion	Exclusion
***Quantitative studies***		
**Population**	• Young people living with HIV (aged 15–24 years)	• Studies were < 50% of the population is between the ages of 15–24 years • Studies that focus on specific population groups (i.e. orphans, LGBTQI, pregnant or post-partum women, sex workers, homeless youth, patients with co-morbidity)
**Study design**	• Observational research study designs or standard of care arm from a trial • Studies that statistically examined factors associated with subjective or psychological wellbeing or any of its dimensions using regression techniques • Studies that statistically examined factors associated with mental health (i.e. mental illness- depression, anxiety) using regression techniques	• Letters, opinion pieces, editorials, reviews, qualitative studies • Psychometric evaluations • Studies were the sample size is *n* < 50
**Outcomes**	• Predictors of any dimension of subjective or psychological wellbeing or mental health	• Outcomes related to physical functioning • Outcomes related to objective measures of quality of life (i.e. birth rate, school completion, mortality)
**Other**	• Peer-reviewed journal articles and non-published studies (conference abstracts, dissertations, working papers) • English and non-English studies • Studies conducted between January 2000–May 2019	
***Qualitative studies***		
**Sample**	• Young people living with HIV (aged 15–24 years) • Caregivers of young people aged 15–24 years, healthcare workers, educators, other family members	• Studies that focus on specific population groups (i.e. orphans, LGBTQI, pregnant or post-partum women, sex workers, homeless youth, patients with co-morbidity)
**Phenomenon of interest**	• Subjective and psychological wellbeing, mental health	• Studies examining objective measures of quality of life
**Design**	• Studies incorporating any form of qualitative study design, data collection method or analytical technique • Cross-sectional or longitudinal	• Studies with YPLHIV in the intervention arm of a trial • Reviews, editorials, letters, essays, theoretical and opinion papers • Studies evaluating a specific policy, programme or intervention
**Evaluation**	• Studies aimed at understanding the lived experiences of wellbeing or experiences related to any dimension of wellbeing or mental health	• Narrow focus on physical functioning, ART adherence, disclosure challenges, sexual reproductive health needs
**Research type**	• Qualitative or mixed-methods	• Quantitative studies
**Other**	• Peer-reviewed journal articles and non-published studies (conference abstracts, dissertations, working papers) • English and non-English studies • Studies conducted between January 2000–May 2019	

#### Studies

We included published and non-published quantitative and qualitative studies. We selected observational quantitative studies reporting on primary or secondary data analysis from cohort or cross-sectional datasets. We deemed any qualitative study design, data collection technique (e.g. group interviews, in-depth interviews, participant observations) or analytical approach (e.g. thematic analysis, framework analysis) eligible for the qualitative arm of this review. Furthermore, we included mixed-method studies that satisfied corresponding inclusion and exclusion criteria, separately determining eligibility for quantitative and qualitative components. In addition, we scanned the bibliographies of relevant quantitative or qualitative reviews to identify potentially eligible primary studies not yielded by the primary search.

#### Participants

We selected studies focused on young people, defined as older adolescents and young adults between the ages of 15–24 years, who were living with HIV [69]. We focused on this condition only as HIV/AIDS remains the leading cause of mortality among young people in this region [70, 71]. We included studies if at least the average age of the sample was within our age-range or results could be extractable for the age-range.

#### Outcome measures

Drawing from the field of social psychology, we conceptualised wellbeing as subjective and psychological. Thus, our outcome measures included any dimension of these two constructs: 1) subjective- positive and negative affect (mood states, emotions), life satisfaction [13]; 2) psychological- self-acceptance, positive relations, environmental mastery, autonomy, purpose in life, personal growth [43, 72]. Given the strong correlation between mental health and wellbeing [21, 72, 73], we included mental health as one of our outcome measures. We applied a biomedical definition of mental health (i.e. the absence or presence of symptoms of mental illness) [74, 75].

We included: quantitative studies that assessed factors associated with any dimension of wellbeing or mental health using statistical regression techniques; qualitative studies that explored lived experiences of wellbeing or experiences related to dimensions of wellbeing or mental health.

#### Study setting

We included studies conducted in any setting (e.g. household, clinic, school) in a country within the sub-Saharan African region, as per the World Bank country classification [76]. We chose to focus on SSA only as this region accounts for the highest number of YPLHIV [77]. Furthermore, young people living in this region have the highest risk of HIV acquisition [78].

#### Time and language

We restricted the search to studies conducted between 01 January 2000 and 11 May 2019. This timeline covers the key post-ART periods in SSA: 1) ART introduction (2000–2007); 2) expanded ART (2008–2010); and 3) scaled-up ART (2011–2019) [79]. We considered the post-ART period more relevant to the current international HIV policy landscape as treatment for all HIV-positive individuals is now the recommended approach [80]. No language restrictions were placed on the search.

### Information sources

Aiming for a broad interdisciplinary approach, we searched published and grey literature on multiple electronic platforms. We searched 6 electronic databases for eligible peer-reviewed journal articles: Medline, Web of Science, PsychINFO, Econlit, Africa-Wide Information, International Bibliography of the Social Sciences. For grey literature, we searched the dissertation databases (Dissertations and Theses- A&I, World Cat), and the International AIDS Society conference archives (2001–2018). In addition, eligible working papers were identified by searching data repositories of the Organisation for Economic Co-operation and Development, IDEAS and the National Bureau of Economic Research.

### Search strategy

We searched electronic databases using either compound search strategies containing subject headings that were supplemented with text terms (Additional files 3, 4, 5 and 6) and JEL codes (Additional file 5), or simple Boolean logic search strategies with keywords (Additional files 7, 8, 9 and 10). Our search terms were aligned to dimensions of the subjective and psychological wellbeing constructs (e.g. negative and positive affect, life satisfaction, self-acceptance, social relationships) [13, 72], with a specific focus on relational dimensions. In addition, our search included terms related to the attitudes and symptoms of common mental disorders (i.e. depression, anxiety) among YPLHIV [81].

### Study records- data management, selection process, data collection process

We imported search outputs into an EndNote (X 8) library and removed duplicate references. Thereafter, we imported this library into an online systematic review manager (Covidence systematic review software, Veritas Health Innovation, Australia). DG and IDA dual screened titles and abstracts independently, and 10% of these were dual screened by GF. AW assisted DG with the dual screening of grey literature. DG subsequently resolved conflicts via discussion with the dual screener, obtained the full text for all potentially eligible abstracts and applied the inclusion criteria to these studies. GF, JS, CM randomly checked all full texts. Less than 5% of abstracts were non-English language studies. For these specific studies, we obtained a translated electronic English version of the study.

### Data items

Once consensus was reached on eligible studies, DG entered relevant data from potentially eligible studies onto electronic data extraction forms. The design of these forms were informed by the Strengthening the Reporting of Observational Studies in Epidemiology (STROBE) checklist [82] and Consolidated criteria for Reporting Qualitative Research (COREQ) checklist [83], for quantitative and qualitative studies, respectively.

We extracted information on the following variables from each included study: author, year published, study setting, country income classification [76], design, outcome definition, analytical techniques, participant demographics, treatment status, disclosure status. For quantitative studies, we specifically extracted data from regression models (e.g. sample size, measures of associations, confidence intervals and *p*-values). For qualitative studies, we extracted primary themes or first order constructs (i.e. participants’ understanding as reported in the study via verbatim quotes or authors description), and secondary themes or second order constructs (i.e. authors’ interpretations of participants’ understandings). Co-authors randomly checked data extractions.

### Outcomes

- Quantitative: explanatory variables associated with wellbeing or mental health, and wellbeing and mental health measures.
- Qualitative: meanings and manifestations of wellbeing, any dimension of wellbeing or mental health

### Quality appraisal

All potentially eligible studies were critically appraised by assessing their methodological quality. For quantitative studies, we applied a risk of bias tool, adapted from the Cochrane guidance on assessing risk of bias in non-randomised studies [67]. DG and GF selected items for this tool based on their relevance to observational study designs in epidemiology and psychology. Studies were categorised into three groups, depending on the level of bias: low, medium or high risk of bias. This was assessed by evaluating measures applied to reduce the following biases: 1) selection bias — random sampling techniques; 2) information bias — training of interviewers in the administration of the scale, translation of the scale to local language, assessing the validity and reliability of scale for given population; 3) confounding — adjustments for potential confounders. For qualitative studies, we applied a quality assessment tool adapted from the COREQ checklist [83] and key studies [84, 85]. DG and JS selected the following indicators for this tool based on its relevance to qualitative research design 1) rigor — use of appropriate theoretical frameworks, sufficient data collected; 2) sincerity — self-reflexivity, transparency about the methods and challenges; 3) credibility — triangulation of data, inductive nature of derived themes.

### Data analysis-synthesis

We conducted a simple descriptive analysis of key quantitative findings. We categorised studies by country income classification and SSA sub-region as the quality of life among YPLHIV may vary depending on the country’s healthcare resources and HIV-epidemic pattern specific to that sub-region. For the qualitative studies, we applied meta-ethnographic analytical methods [86], as implemented by Atkins, Lewin [87]. We specifically used the reciprocal translation analytical approach to develop themes. This approach entailed analysing and synthesising participant views (first-order construct) and authors’ interpretation (second order constructs) to develop third-order constructs. We implemented this approach by comparing first and second order constructs across studies that were homogeneous in terms of design elements (setting, population and period of ART roll-out) and chronologically ordered studies based on publication date [87]. Subsequently, we matched themes across papers ensuring the third order captured similar themes from various studies. We then tabulated translations by highlighting key third order constructs derived and supporting quotes and narratives. Finally, we interpreted the themes across studies to develop a line-of-argument synthesis describing how all themes interacted to shape wellbeing. We evaluated the quality of the qualitative evidence synthesis using the GRADE-CERQual (Confidence in Evidence from Reviews of Qualitative research) approach [88]. This approach includes an assessment of the methodological limitations, coherence, relevance, and adequacy. Lastly, we integrated the main quantitative and qualitative findings by mapping key correlates and themes that emerged from the data to subjective and psychological wellbeing dimensions [13, 72]. Thereafter, we interpreted our data drawing on the relationality meaning model [60].

## Results

### Screening protocol

**Fig. 1:**
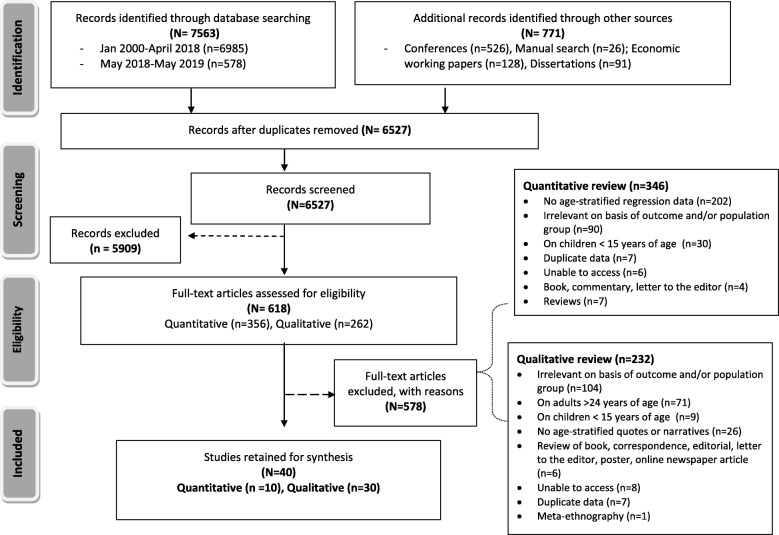
Selection process for the inclusion of studies

The electronic database search yielded a total of 7563 citations, and our grey literature and manual search yielded 771 citations (Fig. 1). After removal of duplicate entries, 6527 studies were evaluated using title and abstracts; 5909 citations were excluded as these were based on populations that were not in line with the reviews’ study population (i.e. caregivers, older adults, elderly, pregnant women) or outcome (i.e. drug effectiveness, access to education or sexual reproductive health services). The remaining 618 potentially eligible studies were retrieved for full-text assessment (356 quantitative review, 262 qualitative review). Of the 356 citations identified for the quantitative review, 10 studies were included, and 346 studies excluded mainly due to the lack of age-stratified analyses or focus on a biomedical outcome measure. Of the 262 citations identified for the qualitative review, 30 studies were included, and 232 studies were excluded mainly because of: 1. a narrow focus on barriers to disclosure or adherence; 2. examined outcomes not related to subjective or psychological wellbeing dimensions (e.g. feasibility and acceptability of a biomedical intervention or health service programme); 3. included adolescents or young adults in the sample but did not provide quotes from participants that spanned the age-range 15–24 years.

### Overall study characteristics

Tables 2 and 3 summarize the main characteristics of the 40 studies (10 quantitative and 30 qualitative). These studies were equally representative of the eastern and southern sub-regions of SSA, with nearly 50% conducted in low-income country settings (*n* = 19). The majority were conducted during the scaled-up ART phase. Participants were mainly between 15 to 19 years of age and sampled from urban public healthcare facilities. None of the included studies defined wellbeing in terms of subjective or psychological wellbeing as defined by Diener and Ryan [89] and Ryff and Keyes [72], respectively. However, these studies examined dimensions related to these constructs such as mental health, relationships and self-acceptance.

**Table 2:** Description of studies in the quantitative synthesis that examined correlates of wellbeing or mental health among YPLHIV in SSA (*N* = 10)

Author year	World Bank Income Classification	Country	Setting	Study design	Data collection period	Type of participants	Recruited from	Outcome measured (scales used)	Total participated (N)	Mean age (SD)	Female n (%)
(Abebe et al., 2019)	Low income (Eastern)	Ethiopia	Addis Ababa (Urban)	Cross-sectional	May-Jun 2016 (Scaled-up ART)	YPLHIV (15–24 yrs.)	Public hospitals	Depressive symptoms (BDI-II)	507	18.6 (3.02)	272 (69)
(Kim et al., 2015)	Low income (Southern)	Malawi	Zomba, Lilongwe (Urban)	Cross-sectional	2012 (Scaled-up ART)	YPLHIV (12–18 yrs.)	Paediatric HIV clinic, ART clinic in hospital	Depressive symptoms (BDI II, CDRS-R)	562	14.5 (2.0)	315 (56)
(Mbalinda et al., 2015)	Low income (Eastern)	Uganda	Eastern, western and northern regions-(Mixed)	Cross-sectional	Sept 2013-Mar 2014 (Scaled-up ART)	YPLHIV (10–19 yrs.), peri-natally HIV-infected	Public and non-profit private healthcare facilities (*n* = 4)	Health-related quality of life (MOS-HIV)	614	16.2 (2.1)	361 (58.8)
(Mutumba et al., 2017)	Low income (Eastern)	Uganda	Kampala (Urban)	Cross-sectional	May- Sept 2013 (Scaled-up ART)	YPLHIV (12–19 yrs.), aware of HIV status, no clinically documented cognitive limitations	NPO ARV Clinic- Joint Clinical Research Centre	Psychological distress (Psychological distress measure)	464	15.6 (2.21)	249 (53)
(Dow et al., 2016)	Low income (Eastern)	Tanzania	Moshi (Urban)	Cross-sectional	Dec 2013-May 2014 (Scaled-up ART)	YPLHIV (12–24 yrs.), aware of HIV status, living with family and attending a HIV- youth programme	HIV youth clinic	Depressive symptoms (PHQ-9), mental health difficulties (SDQ)	182	17.2 (2.9)	99 (54)
(Gaitho et al., 2018)	Lower middle income (Eastern)	Kenya	Nairobi (Urban)	Cross-sectional	Aug-Dec 2016 (Scaled-up ART)	YPLHIV (10–19 yrs.)	Comprehensive Care Clinic in Hospital	Depressive symptoms (PHQ-9)	270	14.75 (2.6)	125 (47.3)
(Okawa et al., 2018)	Lower middle income (Southern)	Zambia	Lusaka (Urban)	Cross-sectional (Mixed-methods)	Apr-Jul 2014 (Scaled-up ART)	YPLHIV (15–19 yrs.), aware of HIV status, registered as clients at the HIV centres	Paediatric and AdultHIV Centres of Excellence -University Teaching Hospital	Depressive symptoms (CES-D)	190	16 (NR)	110 (57.9)
(Gentz et al., 2017)	Upper middle income (Southern)	Namibia	Katutura, Windhoek (Peri-urban)	Cross-sectional	July 2013-Mar 2014 (Scaled-up ART)	YPLHIV (12–18 yrs.), aware of HIV status	Paediatric ARV clinic in hospital	Mental health difficulties (SDQ)	99	14.3 (1.8)	52 (52.5)
(Earnshaw et al., 2018)	Upper middle income (Southern)	South Africa	Johannesburg (Peri-urban)	Cross-sectional	Nov 2015-Jul 2016 (Scaled-up ART)	YPLHIV (13–24 yrs.), aware of HIV status, peri-natal HIV-infection	Paediatric Wellness Clinic- in hospital	Depressive symptoms (BDI-II)	250	16.34 (2.67)	103 (41)
(Woollett et al., 2017)	Upper middle income (Southern)	South Africa	Johannesburg (Urban)	Cross-sectional	Aug 2013-April 2014 (Scaled-up ART)	YPLHIV (13–19 yrs.)	Paediatric clinics- hospital (*n* = 3), community healthcare centre (*n* = 1); primary healthcare clinic (*n* = 1)	Depressive symptoms (CDI-S), anxiety symptoms (RCMAS-2)	343	16* (IQR 12–19)	181 (52)

*SD* Standard Deviation; *PHQ-9* Patient Health Questionnaire 9; *SDQ* Strengths and Difficulties Questionnaire, *BDI II* Beck Depression Inventory-II, *CDRS-R* Children’s Depression Rating Scale–Revised, *MOS-HIV* HIV Medical Outcomes Survey, *CES-D* Center for Epidemiologic Studies Depression Scale, *CDI-S* Children’s Depression Inventory-Short Version, *RCMAS-2* Revised Children’s Manifest Anxiety Scale - Second Edition

**Table 3:** Description of studies included in the qualitative synthesis (*N* = 30). These studies examined lived experiences related to wellbeing or mental health among YPLHIV in SSA

Reference	Income level (sub-region)	Country	Setting (location)	Data collection period	Aim/s of the study	Participant population	Recruited from (sampling strategy)	Data collection method and analysis type
(Bernays et al., 2017)	Low income (Eastern)	Uganda, UK, Ireland, USA	Urban (Kampala)	Scaled-up ART (2011–2015)	To investigate young people’s perspectives on the social and relational challenges encountered in treatment adherence	▪ YPLHIV-Ugandan sample (*n* = 26, 11–22 yrs., mean age 16, F = 14, M = 12)	Healthcare facility (Convenience and purposive)	• 26 IDIs, 2 follow-up IDIs (3 IDIs per participant), semi-structured • Thematic analysis, using a grounded approach and systematic case comparison
(Dusabe-Richards et al., 2016)	Low income (Eastern)	Uganda	Rural (South-Western, Kalungu district)	Scaled-up ART (2011–2012)	To understand the communication challenges of disclosure and its aftermath within these relationships from the dual perspectives of the older carer and the HIV positive child in their care	▪ YPLHIV (*n* = 18, 13–17 yrs., F = 8, M = 10 ▪ Older caregivers (*n* = 18)	Healthcare facility (Convenience)	• 8 IDIs YPLHIV, 18 IDIs caregivers, semi-structured • Thematic analysis
(Inzaule et al., 2016)	Low income (Eastern)	Uganda	Urban (Kampala, Fort Portal, Mbale)	Scaled-up ART (May-Aug 2015)	To assess the challenges to long-term adherence in adolescents and adults in three regional HIV treatment centres in Uganda	▪ Expert adolescent clients (*n* = 5, age-NR, sex-NR); ▪ HCWs (*n* = 28)	Healthcare facility (Purposive)	• 24 IDIs, 2 FGDs, semi-structured • Thematic analysis
(Kajubi et al., 2016)	Low income (Eastern)	Uganda	Coastal (Jinja district-Lake Victoria)	Expanded ART (Nov 2011-Dec 2012)	To explore the implications of different family constellations for caregiving and communication with children on ART	▪ YPLHIV (*n* = 29; 8–17 yrs., mean age 12 yrs., F = 16, M = 13)	Healthcare facility (Purposive)	• 29 Participant observations with follow-up for 12 months, and 29 IDIs, semi-structured • Thematic analysis
(Knizek et al., 2017)	Low income (Eastern)	Uganda	Mixed-urban and semi-urban/rural (Kampala, Masaka)	Scaled-up ART (Jul-Nov 2015)	To investigate both the protective and the risk factors in HIV-infected adolescents’ care environment in order to understand what might contribute to negative outcomes and what might provide a protective buffer against harmful life events	▪ YPLHIV (*n* = 21, 12–17 yrs., mean age 14.6 yrs., F = 12, M = 9)	Healthcare facility (Convenience and purposively sampled)	• 21 IDIs with vignettes, semi-structured • Phenomenological approach
(Kyaddondo et al., 2013)	Low income (Eastern)	Uganda	Mixed-urban, peri-urban, rural (Kampala, Mpigi, and Soroti districts)	Expanded ART (May 2008-Sept 2009)	To examine the moral dilemmas and pragmatic incentives surrounding disclosure of HIV status in contemporary Uganda	▪ PLHIV (*n* = 12, 6 aged 18–24 yrs., F=NR, M = NR)	Healthcare facility (Convenience)	• 12 IDIs (6 YPLHIV), 2 FGDs (YPLHIV NR), 6 key informant interviews • Method of analysis NR
(Loos et al., 2013)	Low income (Eastern)	Uganda	Mixed- urban and rural (Kampala, Kisumu, Kamito and Wagai)	Expanded ART (Jul-Nov 2009)	To assess the impact of HIV and related contextual conditions on identity formation of adolescents living with HIV/AIDS (ALH) in the domains of physical, cognitive, social, and sexual development	▪ YPLHIV (*n* = 119,10–19 yrs., mean age 13.5 yrs., F = 64, M = 55) ▪ Caregivers (*n* = 6) ▪ HCWs (*n* = 53)	Healthcare facility (Convenience)	• 16 FGDs (YPLHIV, stratified by age and sex- 10-12, 13–15, 16–19 yrs.); 6 FGDs (caregivers), 6 FGDs (HCWs), semi-structured • Thematic analysis
(Mathur et al., 2016)	Low income (Eastern)	Uganda	Rural (Rakai)	Expanded ART (Jun 2010-Jul 2011)	To examine relationship and life events to hopefully describe some of the circumstances that influenced young men’s HIV vulnerability and acquisition	▪ YPLHIV and their HIV-negative partners (*n* = 30, 15–24 yrs., mean age 22 yrs., F = 0, M = 30)	Community (Purposive)	• 30 IDIs- life history interviews, semi-structured • Thematic analysis
(Matovu et al., 2012)	Low income (Eastern)	Uganda	Urban (Kampala)	Expanded ART (Jan-Feb 2009)	To explore how young women with HIV/AIDS in Uganda experience the influence of their everyday life occupations on adherence to HAART after more than 1 year on the medication	▪ YPLHIV (*n* = 6, 16–20 yrs., F = 6, M = 0)	Healthcare facility (Purposive)	• 6 narratives, 2 interviews per participant conducted over 1 month, semi-structured • Thematic analysis
(Mutumba et al., 2015)	Low income (Eastern)	Uganda	Urban (Kampala)	Scaled-up ART (Aug-Nov 2011)	To identify the psychosocial challenges and coping strategies among perinatal HIV-infected adolescents in Uganda	▪ YPLHIV (*n* = 38, 12–19 yrs., mean age 16.9 yrs., F = 20, M = 18)	Healthcare facility- clinical research centre (Purposive)	• 38 IDIs, semi-structured • Thematic analysis- grounded in a phenomenological approach
(Siu et al., 2012)	Low income (Eastern)	Uganda	Urban (Kampala)	Expanded ART (May-Jun 2009)	To describe HIV serostatus and treatment disclosure practices and concerns from the perspective of YPLHA in Uganda, exploring their satisfaction with current norms around HIV serostatus and treatment disclosure- examines disclosure and lived experiences	▪ YPLHIV (*N* = 20, 15–23 yrs., median age 20 yrs., F = 10. M = 10)	Healthcare facility (Purposive)	• 20 IDIs, 2 FGDs (sex-disaggregated), field notes, semi-structured • Thematic analysis
(Mattes, 2014)	Low income (Eastern)	Tanzania	Coastal (North-eastern, Tanga city)	Expanded ART (Sept 2008-Sept 2011)	To compare the national guidelines’ imaginary versions of HIV disclosure and treatment management with the lived realities of paediatric HIV management in a specific north-eastern Tanzanian Care and Treatment Centre (CTC) and in affected children’s social environments	▪ YPLHIV (*n* = 13, 9–19 yrs., F = 5, M = 8) ▪ Caregivers (*n* = 11)	Healthcare facility (Convenience)	• 13 IDIs with thematic drawings, participant observations (YPLHIV); Caregivers (NR), semi-structured • Grounded theory approach
(Abubakar et al., 2016)	Lower middle income (Eastern)	Kenya	Coastal (Kilifi)	Scaled-up ART (2012–2013)	To investigate the experiences and challenges of HIV infected adolescents at the Kenyan coast	▪ YPLHIV (*n* = 12, 12–17 yrs., mean age 14.5 yrs., F = 3, M = 9); ▪ HIV uninfected (*n* = 7, 12–17 yrs., mean age = 15 yrs., F = 5, M = 2); ▪ Caregivers (*n* = 11) ▪ HCWs& CHWs (*n* = 8) ▪ Educators (*n* = 6)	Healthcare facility- YPLHIV, caregivers, HCWs, CHWs; Community-HIV uninfected, Secondary schools- educators (sampling strategy = NR)	• 30 KIIs, semi-structured • Framework approach
(Adegoke and Steyn, 2017)	Lower middle income (Western)	Nigeria	Urban (Ibadan city- Oyo state)	Scaled-up ART (2013)	To explore the experiences of Yoruba adolescent girls living with HIV, particularly factors contributing to their resilience	▪ YPLHIV (*n* = 5, 20 yrs., mean ag 20 yrs., F = 5, M = 0)	Community NGO (Purposive)	• 5 Photo-voice coupled with narratives (participatory action research) • Secondary inductive content analysis
(Campbell et al., 2012)	Low middle income (Southern)	Zimbabwe	Rural (Manicaland)	Expanded ART (Oct 2009-Mar 2010)	To investigate the social landscape of children’s adherence in rural Zimbabwe through	▪ Caregivers (*n* = 40) ▪ Nurses (*n* = 25)	Healthcare facility: (snowball, self-selected informants, typical case -caregivers, convenience-nurses)	• 39 IDIs, 3 FGDs • Thematic network analysis
(Lypen et al., 2015)	Lower middle income (Eastern)	Kenya	Urban (informal settlement) (Kibera- Nairobi)	Expanded ART *(NR)	To better understand the complex social support systems among these youth as well as this support’s influence on their HIV management and related coping mechanisms	▪ YPLHIV (*n* = 53, 18–27 yrs., mean age 22.8, F = 26, M = 27)	Healthcare facility (Modified respondent driven sampling)	• 6 FGDs (stratified by sex) • Phenomenological approach
(Mburu et al., 2014)	Lower middle income (Southern)	Zambia	Mixed- rural and urban (Kalomo, Kitwe, Lusaka)	Expanded ART (Apr-Dec 2010)	To document the experiences of adolescents living with HIV with regard to disclosure, specifically addressing: adolescents who were previously unaware of their HIV-positive status being told about it by their parents, and adolescents who know about their HIV-positive status telling others about it	▪ YPLHIV (*n* = 58, 10–19 yrs., mean age 16.8 yrs., F = 29, M = 29) ▪ Caregivers (*n* = 21) ▪ HCWs (*n* = 14)	Healthcare facility, community and youth centres (Convenience)	• 8 FGDs, 58 IDIs (YPLHIV); 2 FGDs (caregivers); 3 FGDs, 14 IDIs (HCWs), semi-structured • Thematic analysis
(Shabalala et al., 2016)	Lower middle income (Southern)	eSwatini (formerly Swaziland)	Mixed- 1 rural, 1 urban (Manzini region)	Scaled-up ART (Jul 2012-Dec 2013)	To explore the meaning of the family as it applies to Swazi adolescents’ everyday life	▪ YPLHIV (*n* = 13, 12–19 yrs., mean age 13.6 yrs., F = 5, M = 8)	Healthcare facility (Convenience)	• 13 IDIs (YPLHIV), FGDs (*n* = NR), KIIs (*n* = NR), semi-structured • Thematic analysis using an inductive approach
(Mackworth-Young et al., 2017)	Lower middle income (Southern)	Zambia	Urban (Lusaka)	Scaled-up ART (Jan-Apr 2015)	To explore the experiences of adolescent girls growing up with HIV in Lusaka, Zambia	▪ YPLHIV (*n* = 24, 15–18), F = 24, M = 0)	Healthcare facility (Convenience)	• 4 participatory workshops (used concept mapping, collages and vignettes); 34 IDIs- 17 interviewed twice, used network tools • Thematic analysis using a grounded theory approach
(Goudge et al., 2009)	Upper middle income (Southern)	South Africa	Urban (Gauteng province)	ART introduction (2006–2008)	To document the diverse journeys of people living with HIV after the national roll-out of ARV treatment, through ill health, testing, disclosure, and treatment, and their responses to stigma	▪ PLHIV (*n* = 5, 20–54 yrs., *n* = 1 20–24 yrs., F = 3, M = 2)	Healthcare facility (Random from an existing survey)	• IDIs with narratives, interviewed twice over 6 months, semi-structured • Narrative approach
(Li et al., 2010)	Upper middle income (Southern)	South Africa	Urban (Tygerberg, Western Cape)	Expanded ART (2009)	To explore the experiences and needs of a group of adolescents living with HIV in Cape Town, South Africa	▪ YPLHIV (*n* = 26, 7–15 yrs., mean age 12.5 yrs., F = 10, M = 16)	Healthcare facility (Convenience)	• 4 FGDs, 26 IDIs, used photographs and pictorial messages, semi-structured • Thematic analysis
(Midtbo et al., 2012)	Upper-middle income, low income (Southern, Eastern)	Botswana, Tanzania	Mixed-Urban and rural	Scaled-up ART (Jun-Sept 2011)	To understand and identify the pathways between HIV-status disclosure, ART, and children’s psychosocial wellbeing, including from the perspective of adolescents themselves	▪ YPLHIV (*n* = 28, 12–20 yrs., F = 17, M = 11); ▪ HCWs (*n* = 3)	Community NGO, hospital (Purposive)	• 2 FGDS, 28 IDIs (YPLHIV); 3 IDIs (HCWs), participant observations, semi-structured • Thematic analysis
(Plattner and Meiring, 2006)	Upper middle income (Southern)	Namibia	Urban (Windhoek)	ART introduction (2003)	To better understand the psychological coping processes from the perspectives of infected people	▪ PLHIV (*n* = 10, 20–48 yrs., F = 8, M = 2)	Community NGO (Convenience)	• 10 IDIs, semi-structured • Circular deconstruction method
(Jena, 2014)	Upper middle income (Southern)	South Africa	Urban (Eastern Cape-Port Elizabeth)	Scaled-up ART (Nov 2013)	To explore the lived experiences of adolescents living with vertically acquired HIV	▪ YPLHIV (*n* = 6, 16–17 yrs., F = 4, M = 2, all vertically HIV-infected)	Healthcare facility (Purposive)	• 6 IDIs- semi-structured • Thematic analysis
(Petersen et al., 2010)	Upper middle income (Southern)	South Africa	Urban (KwaZulu-Natal-Durban)	Expanded ART (2008)	To examine the psychosocial challenges and protective factors for adolescents and their caregivers affected by paediatric HIV within the sociocultural context of South Africa	▪ YPLHIV (*n* = 25, 14–16 yrs. F=NR, M = NR) ▪ Caregivers n-15)	Healthcare facility (Purposive)	• 25 IDIs • Thematic analysis
(Pienaar and Visser, 2012)	Upper middle income (Southern)	South Africa	Urban (Gauteng-Pretoria)	Expanded ART (2010)	To describe the experiences of the adolescent who live with HIV and undergo chronic disease management at the Kalafong Paediatric HIV clinic, so as to gain an understanding of the meanings they attribute to their experiences of HIV that informs their identities	▪ YPLHIV (*n* = 6, 13–17 yrs., F = 3, M = 3)	Healthcare facility (Purposive)	• 6 IDIs with follow-up interviews-semi-structured with drawings and storytelling • Narrative analysis
(Rosenbaum, 2017)	Upper middle income (Southern)	South Africa	Peri-urban (Katlehong Township- Gauteng province)	Scaled-up ART* (NR)	To develop a cultural understanding of how young people living with HIV effectively cope with the adversities that they face and the social ecological resources that contribute to their well-being and resilience	▪ YPLHIV (*n* = 7, 17–19 yrs., mean age 18 yrs., F = 2, M = 5); ▪ Mental healthcare provides (*n* = 3)	Clinic support group (Purposive)	• 7 FGDs with photo-voice (YPLHIV), interviews (mental healthcare providers), semi-structured • Thematic analysis
(Vale et al., 2017)	Upper middle income (Southern)	South Africa	Mixed-rural and peri-urban (Eastern Cape- rural village (Mtembu) and peri-urban informal settlement (Ridgetown))	Scaled-up ART (Aug-Dec 2013, Jan-April 2014)	To understand how tacit inferences about adolescents’ mode of infection contribute to their experiences of HIV-related blame, and their ability to achieve care, in their intimate, everyday settings	▪ YPLHIV (*n* = 23, 10–19 yrs., F = 23, M = 0); ▪ Caregivers (*n* = NR)	Community NGO (Purposive)	• 20 IDIs- YPLHIV and mothers, field notes • Narrative approach
(Woollett et al., 2016)	Upper middle income (Southern)	South Africa	Urban (Johannesburg)	Scaled-up ART (Oct 2014-Nov 2015)	To identify elements of resilience in a group of perinatally infected HIV positive adolescents attending HIV clinics	▪ YPLHIV (*N* = 25, 13–19 yrs., F = 15, M = 10)	Healthcare facility (Purposive)	• 25 IDIs, semi-structured • Thematic analysis
(Woollett et al., 2017)	Upper middle income (Southern)	South Africa	Urban (Johannesburg)	Scaled-up ART (Aug 2013- April 2014)	To examine the perceptions of perinatally infected HIV-positive adolescents attending clinics in Johannesburg with respect to their own infection, how they were disclosed to and their mental health state	▪ YPLHIV (*n* = 25, 13–19 yrs., mean age 16 yrs., F = 15, M = 10)	Healthcare facility (Purposive)	• 25 IDIs, semi-structured • Thematic analysis

*NR* not reported

### Quantitative studies-characteristics and data synthesis

Of the ten quantitative studies (Table 2), five were conducted in southern Africa: South Africa [90, 91], Namibia [92], Malawi [93], Zambia [94] (Table 3). The remainder conducted in eastern Africa: Uganda (*n* = 2) [95, 96], Kenya (*n* = 1) [97], Ethiopia (*n* = 1) [98] and Tanzania (*n* = 1) [99]. All studies employed a cross-sectional study design. Neither subjective nor psychological wellbeing were measured in any of these studies. All studies measured mental health, specifically symptoms of depression, mainly using the Beck Depression Inventory-II (*n* = 3), Patient Health Questionnaire-9 (*n* = 2), Strengths and Difficulties Questionnaire (*n* = 2). The key factors associated with mental health outcomes measured are detailed below (Table 4, Additional file 11).

i. ***Demographics***

**Table 4:** Results from studies included in the quantitative synthesis (*N* = 10)- correlates associated with wellbeing or mental health among YPLHIV in SSA

Author year	Regression technique	Outcome 1 (scale)	Univariable/Bivariable analysis (effect size, 95% CI, ***p*** value)^**$**^	Multivariable analysis (effect size, 95% CI, ***p*** values) ^**$**^
(Abebe et al., 2019)	Logistic regression	Depressive symptoms (BDI-II)	• 15–19 yrs. (OR = 2.84, 95% CI 1.92–4.21, p ≤ 0.2) • Opportunistic infection (OR = 1.89, 95% CI 1.29–2.78, p ≤ 0.2) • Stigma (OR = 2.74, 95% CI 1.88–4.00, *p* ≤ 0.2) • Poor adherence (OR = 2.11, 95% CI 1.44–3.09, p ≤ 0.2) • Low adherence (OR = 3.22, 95% CI 1.78–5.82, p ≤ 0.2) • Moderate social support (OR = 2.08, 95% CI 1.27–3.39, p ≤ 0.2)	• 15–19 yrs. (OR = 2.20, 95% CI 1.33–3.62, p < 0.01) • Opportunistic infection (OR = 1.94, 95% CI 1.15–3.27, *p* < 0.01) • Stigma (OR = 2.06, 95% CI 1.35–3.14, *p* < 0.001) • Poor Adherence (OR = 1.73, 95% CI 1.13–2.64, *p* < 0.01) • Low social support (OR = 2.74, 95% CI 1.42–5.27, p < 0.01) • Moderate social support (OR = 1.75, 95% CI 1.03–2.98, p < 0.05)
(Dow et al., 2016)	Negativebinomial regression	Depressive symptoms (PHQ-9)	• Age (per 1 year) (MR: 1.12, 95% CI 1.05–1.18, p < .001) • Female (MR: 1.62, 95% CI1.15–2.28; *p* = .006) • Not in school (MR: 1.65, 95% CI 1.12–2.43; p = .01) • Stigma (per 1 point) • (MR: 1.09, 95% CI1.06–1.13; *p* < .001)	• Age (per 1 year) (MR: 1.08, 95% CI 1.03–1.14, *p* = .004) • Female (MR: 1.52, 95% CI1.11–2.09; *p* = .01) • Stigma (per 1 point) (MR: 1.08, 95% CI1.04–1.11; p < .001) • Incomplete adherence (MR: 1.52, 95% CI1.07–2.18; *p* = .02)
(Earnshaw et al., 2018)	Poisson regression	Depressive symptoms (BDI-II)	• Internalised stigma (RR = 1.27, 95% CI 1.19–1.34, p ≤ 0.05) • Associative stigma (RR = 1.55, 95% CI 1.43–1.68, *p* ≤ 0.05) • Internalised*associative stigma (RR = 1.12 (95% CI 1.09–1.14), *p* ≤ 0.05)	• Internalised stigma (RR = 1.23, 95% CI 1.13–1.34, p ≤ 0.05) • Associative stigma (RR = 1.59, 95% CI 1.37–1.84, p ≤ 0.05)
(Gaitho et al., 2018)	Linear regression	Depressive symptoms	• 15–19 years (OR = 2.6, 95% CI 1.6–4.3, *p* < 0.001) • frequent changing of schools in the preceding 2 years due to repeated adversities (OR = 1.66, 95% CI 0.99–2.81, p = 0.05) • repeating a grade (OR = 1.85, 95% CI 1.11–3.11, *p* = 0.02) • lack of school fees (OR = 2.01, 95% CI 1.23–6.31, *p* = 0.005) • unavailability of food (OR = 2.83, 95% CI 1.27–6.31, *p* = 0.009) • ran away from home (OR = 3.39, 95% CI 1.09–10.58, p = 0.03) • substance use (OR = 3.57, 95% CI 1.29–9.92, *p* = 0.01) • non-perfect adherence to their medications (OR = 2.62, 95% CI 1.60,-4.28, p ≤ 0.001)	• 15–19 years (OR = 2.34, 95% CI 1.36–4.04, *p* < 0.02) • having had an experience of repeating a grade (OR = 1.74, 95% CI 1.0–3.05, *p* = 0.05) • having had an experience of being refused school participationdue to lack of school fees (OR = 1.71, 95% CI 1.0–2.91, p = 0.05) • non-adherence to medication (OR = 1.84, 95% CI 1.08–3.14, *p* = 0.03)
(Gentz et al., 2017)	Hierarchical multiple linear regression	Total difficulties-(SDQ)	*Total difficulties* • Orphanhood • (β = 0.138, 95% CI NR, *p* < .05	*Total difficulties-Final model* • Child assets (β = − 0.22, 95% CI NR, *p* < 0.05) • Stigma • (β = − 0.261, 95% CI NR, p < 0.05)
(Kim et al., 2015)	Linear/ logistic regression	Depressive symptoms (BDI II)	NR	Final model • Female (β: 2.13, 95% CI 0.82–3.43, p = 0.002) • Not in school/junior primary (β: 3.84, 95% CI 1.71–5.98, *p* = 0.0005) • Nobody in my family has died (β: − 1.77, 95% CI − 3.15- − 0.39, *p* = 0.001) • Did not fail school term/class (β: − 1.46, 95% CI − 2.76- − 0.17, *p* = 0.003) • Bullying for taking medication (β: 5.31, 95% CI 3.19–7.43, *p* < 0.0001) • Never had a boyfriend/girlfriend (β: − 2.38, 95% CI − 4.35- − 0.41, *p* = 0.02) • Disclosed and have shared with someone (β: − 1.83, 95% CI − 3.79-0.13, p = 0.02) • Level of immunosuppression (None or not significant) (β:− 2.58, 95% CI − 4.29- − 0.87, *p* = 0.0009) • Age* satisfaction with physical appearance interaction (β:− 0.93, 95% CI − 1.74- − 0.11, p = 0.03) • Age* Height for age z-score interaction –(β: − 0.39, 95% CI − 0.68- − 0.11, *p* = 0.007)
(Mbalinda et al., 2015)	Logistic regression	Physical health functioning-(MOS- HIV)	NR	• Secondary (aOR: 0.41, 95% CI 0.20–0.85, p = 0.01) • Northern region (aOR: 0.25, 95% CI0.16–0.42; p = < 0.001) • Currently on ARVs (aOR: 2.07, 95% CI1.24–3.36; p < 0.05) • Has a friend who is smoking cigarette- (aOR: 0.48, 95% CI0.29–0.80; p = < 0.001)
(Mutumba et al., 2017)	Hierarchical multiple linear regression	Psychological distress	NR	Final model • Female (β: 0.061, 95% CI NR, *p* = 0.08) • Pentecostal (β: 0.086, 95% CI NR, p = 0.02) • Paternal orphan (β: − 0.083, 95% CI NR, p = 0.05) • Double orphan (β: − 0.094, 95% CI NR, *p* = 0.07) • Daily hassles (β: 0.118, 95% CI NR, *p* = 0.01) • Negative life events (β: 0.209, 95% CI NR, p < 0.01) • HIV-related QoL (β: 0.299, 95% CI NR, p < 0.01) • HIV stigma (β:0.089, 95% CI NR, p = 0.02) • Religiosity (β: 0.078, 95% CI NR, p = 0.02) • Religious coping (β: − 0.083, 95% CI NR, p = 0.02) • Optimism (β:− 0.063, 95% CI NR, *p* = 0.09) • Satisfied with social support (β: − 0.169, 95% CI NR, p < 0.01) • General coping styleand behaviours (β: − 0.160, 95% CI NR, p < 0.01)
(Okawa et al., 2018)	Logistic regression (multiple)	Depressive symptoms (CES-D)		• Fair/unsatisfied with relationship with family (aOR: 3.01, 95% CI 1.20–7.56, p < 0.01) • Fair/unsatisfied with relationship with HCWs (aOR: 2.68, 95% CI1.04–6.93; p = < 0.001) • Experienced HIV stigma (aOR: 2.99, 95% CI1.07–8.41; p = 0.01)
(Woollett et al., 2017)	No formal regression, calculated relative risks using Altman’s formula	Depressive symptoms (CDI-S)	• Been hit (RR: 1.97, 95% CI NR, p 0.02) • Been inappropriately touched (RR: 2.22, 95% CI NR; p = 0.01) • Do not feel like they control their future (RR: 2.55, 95% CI NR; *p* = 0.04) • Do not feel safe at home (RR: 5.17, 95% CI NR; p < .001) • Do not have a dream (RR: 4.62, 95% CI NR; p < .001 • Do not have a safe place in the communityfor adolescents (RR: 2.31, 95% CI NR; p < .001) • Experienced forced sex (RR:3.55, 95% CI NR; p = 0.02) • Experienced peer violence outside of school (RR:2.16, 95% CI NR; p = 0.01) • Experienced peer violence at school andoutside (RR:1.77, 95% CI NR; p = 0.04) • Reports any form of suicidality (RR: 3.44, 95% CI NR; p < .001) • Think about a way to kill themselves (RR: 3.54, 95% CI NR; p < .001) • Think about killing themselves (RR: 3.22, 95% CI NR; p < .001) • Try to kill themselves- (RR: 3.76, 95% CI NR; p < .001) • Want to hurt themselves- (RR: 2.74, 95% CI NR; p < .001) • Wish they were dead- (RR: 3.71, 95% CI NR; p < .001)	• NR

*NR* Not reported, *MR* Mean ratio, *OR* Odds ratio, *aOR* adjusted odds ratio, *CI* Confidence interval, *QoL* quality of life, *RR* risk ratio, $ = factors considered statistically significant (as per the study’s definition) are only presented, * = interaction terms

Being female was strongly correlated with poor mental health functioning in four studies (note, the outcome definition was different in each of the four studies) [92, 93, 96, 99]. The largest gender effect was observed in Malawi, with females almost eight times as likely compared to males to exhibit higher depression-related symptoms scores (β = 2.13, 95% CI [0.82–3.43], *p* = 0.002) [93]. In two urban studies from low income settings, there was between an 8 to 23% increase in depression scores with age [93, 99].

ii. ***Standard of living***

Educational attainment was found to have a protective effect on mental health functioning in three low income countries [93, 95, 99]. In the Ugandan study, those with secondary school attainment were five times more likely to have better mental health than those with no education (adjusted odd ratio (aOR) = 5.3, 95% CI [1.86–15.41], *p* < 0.00) [95].

iii. ***Psycho-social***

HIV-related stigma was strongly positively associated with poor mental health functioning in six studies [91, 92, 94, 96, 98, 99]. Among these studies, the largest effect of stigma was documented in Zambia, which found that the odds of having higher depressive symptom scores was almost three times higher for YPLHIV who experienced stigma than in those who did not (aOR = 2.99; 95% CI [1.07–8.41], *p* = 0.01) [94]. Having someone to talk to or feeling satisfied with health services or the social support received promoted positive mental health functioning in four studies (Namibia (*n* = 1) [92], Uganda (*n* = 2) [95, 96], Zambia (*n* = 1) [94]. In an Ethiopian study, Abebe, Shumet [98] reported that those with low social support were 2.74 times more likely to develop depressive symptoms than those with strong social support (95% CI [1.42–5.27], *p* = < 0.01). Moreover, poor adherence was positively correlated with depressive symptoms in three studies [97–99].

#### Quality of studies

Five of the ten quantitative studies were judged as having low quality as findings were subject to a high risk of bias [90–92, 94, 99] (Table 5). The main quality concerns in these studies were the lack of reporting on psychometric properties of the scale and standardisation of scale administration, including the inadequate reporting and interpretation of statistical analyses. Only one study psychometrically evaluated the chosen scale on a similar study population and found it to have good validity and reliability [93]. None of the studies reported information on the cultural validity of the chosen scale/s. All studies were subject to selection bias as samples were drawn from healthcare facilities, primarily using non-random sampling techniques.

**Table 5:** Quality assessment of studies included in the quantitative synthesis (*N* = 10). These studies examined correlates of wellbeing or mental health among YPLHIV in SSA ^$^

Author year	Sampling (max score 4)		Measurement (max score 8)		Reporting (max score 8)			Total score	Quality
	Generalisability	Sample size	Psychometric properties of scale	Scale administration	Description of analysis	Reporting of regression analysis	Adjustment of confounders	Maximum score = 20	
(Abebe et al., 2019)	1	2	1	3	1	3	1	12	Medium
(Dow et al., 2016)	1	2	1	2	1	1	0	8	Low
(Earnshaw et al., 2018)	1	1	0	1	2	3	1	9	Low
(Gaitho et al., 2018)	1	1	0	1	1	4	2	10	High
(Gentz et al., 2017)	1	0	1	2	0	1	2	7	Low
(Kim et al., 2015)	1	2	3	2	2	3	2	15	Medium
(Mbalinda et al., 2015)	1	2	2	1	1	3	2	12	Medium
(Mutumba et al., 2017)	1	2	2	2	1	3	1	12	Medium
(Okawa et al., 2018)	1	2	1	0	1	1	1	7	Low
(Woollett et al., 2017)	1	2	1	1	0	0	0	5	Low

$ = The quality assessment tool that was applied was adapted from the Cochrane guidance on assessing risk of bias in non-randomised studies (Higgins and Green, 2011)

### Qualitative studies- characteristics and data synthesis

Of the 30 qualitative studies (Table 3), most were undertaken in the eastern sub-region (*N* = 14): Uganda (*n* = 11), Kenya (*n* = 2), Tanzania (*n* = 1) and the southern sub-region (*N* = 14) (South Africa (*n* = 9), Namibia (*n* = 1), Swaziland (*n* = 1), Zambia (*n* = 2), Zimbabwe (*n* = 1), with one study conducted in both regions (Tanzania and Botswana). The remaining study was conducted in West Africa (Nigeria *n* = 1). Only six studies focused on a specific gender (females *n* = 5, males *n* = 1). Whilst none of the 30 studies specifically aimed to examine lived experiences of subjective or psychological wellbeing they did, however, explore experiences related to dimensions of PWB or mental health. The aim of most of the studies was to understand the psycho-social challenges experienced among YPLHIV. Five studies examined broader life experiences associated with wellbeing using ethnographic methods. Data were collected mainly via focus-group discussions and in-depth interviews. Several studies included perspectives from caregivers (*n* = 9) and healthcare workers (HCWs) (*n* = 7), with only one study that included perspectives from educators.

#### Specific analytical themes

**Fig. 2:**
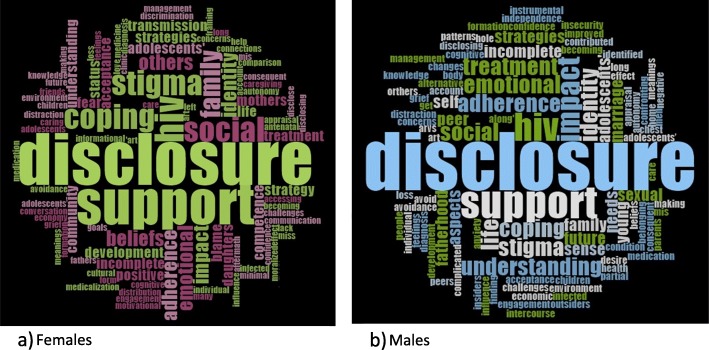
Words and phrases in themes used by authors of studies included in the qualitative synthesis. These studies examined lived experiences related to dimensions of wellbeing or mental health among YPLHIV in SSA. The text font size indicates frequency

Three key themes emerged across all studies: 1) acceptance and belonging, 2) coping; 3) standard of living (Table 6, Fig. 2). These themes shaped experiences suggestive of wellbeing as detailed below.

i. **Acceptance and belonging**

- ***HIV-related stigma and discrimination***

**Table 6:** Key themes that shaped experiences suggestive of wellbeing among YPLHIV in SSA, as derived from the qualitative meta-synthesis

Third order labels	Third order constructs	Second order constructs (authors interpretation)	First order (sample of quotes or narratives)
**Theme 1: Social acceptance and belonging**			
***1.1 HIV-related stigma and discrimination***	o Stigma compromised wellbeing via several pathways o Impact of internalised stigma on identity, social interactions and engagement, medical adherence and mental health functioning o Experienced stigma encountered at various socio-ecological levels exacerbated feelings of isolation and rejection o Internalised and experienced stigma intersected with gender and cultural norms o Stigma reduced feelings of social acceptance and social connectedness o Stigma challenged ability to maintain relationships	- Fear if HIV-positive status was known among the wider community - Caregivers fears on adolescent’s risk for rejection, isolation and stigmatisation	▪ “I’ve thought about telling them [my friends], but then I stop myself because I’m afraid they’ll be mean to me or they’ll mistreat me or they’ll avoid me.” [15 year-old male, South Africa] (Li et al., 2010) ▪ “I think that if I tell other children, they might end up treating him badly or have negative attitudes towards him.” [Grandmother-caregiver, Kenya] (Abubakar et al., 2016)
		- Strategies to prevent unintentional disclosure-keeping one’s status a secret	▪ “After learning of her daughter’s HIV diagnosis, Nandipha’s mother reportedly felt ashamed, suggesting that she perceived the diagnosis to also be a reflection on her. To protect themselves from gossip, the family continued to keep Nandipha’s status a secret” [15–19 year-old, South Africa] (Vale et al., 2017) ▪ “Even at home the children don’t know. They see me and ask but mum tells them I have malaria and they don’t care. Mum tells me not to tell them.maybe in the future.” [18 year-old female, Uganda] (Mutumba et al., 2015)
		- Development of negative identities	▪ “Up to now, I feel different from other children. Someone who looks miserable without HIV is far better than a person who looks healthy with HIV. [Who told you?] It’s how I know it and I believe it’s true” [17 year-old female, Uganda] (Mutumba et al., 2015) ▪ “Oh look at that girl who has AIDS”. I did rather people see me as Musa than them saying “Oh Musa with AIDS” (16–17 year-old male, South Africa) (Jena, 2014)
		- Fears related to infecting sexual partner	▪ “So far I am not thinking about having a girlfriend. […] The problem have is if I infect my partner, does that not even cause more problems? I don’t want to infect my partner the way I was infected. So I think it’s better to calm down and wait for the day that a solution will be available.” [16 year-old male, Tanzania] (Mattes, 2014) ▪ “I have a boyfriend, but I cannot tell him am positive, although he says he loves me and this is stressing me a lot because, I want to get married, but I cannot because he will fall sick and I love him, yet I cannot tell him am positive ….” [20 year-old female, Uganda] (Matovu et al., 2012)
		- Non-disclosure to parents- fear of loss of rights and entitlements	▪ “For me my father is alive and I am the heir, but if he knows that I am positive he might remove the heirship from me thinking that I will die before him. I must first weigh the possible outcome of disclosing and to whom.” [20–24 year-old male, Uganda] (Kyaddondo et al., 2013)
		- Stigma experienced by family members and its consequences- feeling unaccepted by family, interference with medical adherence	▪ “(...) I grew up when my mother never saw me as a person who can really achieve something in future, because I am the only kid who was born HIV positive. (...) So, she saw me like a failure, I would not succeed in anything. (...) She used to discriminate me among my brothers and sisters. She used to treat them as children, but me as nothing. A bastard at home. “(…) I got to know that mothers are the most creatures that really love their children compared to their dads. (…) But I was really surprised that it’s my dad who loves me more than my mum. So I would ask myself why my mother was doing such. At times I would tell myself that this world is nothing for me.” [17 year-old female, Uganda] (Knizek et al., 2017) ▪ “He did not see eye to eye with his sister-in-law who did not like the fact that Mpendulo was HIV positive...in one incidence the sister-in-law found him eating food from a plate that was not designated for him. She scolded the boy for using the plate; stating that...he will infect her children with HIV. That angered Mpendulo a lot. He said he felt unwelcomed and not wanted.” [Case study of 15 year-old male”, eSwatini (formerly Swaziland)] (Shabalala et al., 2016) ▪ “My auntie told me that I do not belong to the family, because of my condition and I was always segregated from other family members. When I go back home my auntie starts throwing insults at me and saying that you have been sleeping around. She doesn’t care, if you tell her please aunt buy for me some clothes, she replies with annoyance that I stopped buying for you clothes in Primary five saying that I no longer have value and I don’t give you my things, it’s up to you. I remember the doctors called her one time to pick my medication and also to act as the adherence support person and she said, if it means for her to die, let it be so, I will not come. I even contemplated killing myself because of the situation” [16 year-old, female, Uganda] (Matovu et al., 2012)
		- Stigma perpetuated by school learners and educators, impact on medical adherence and mental health - Perceived lack of sympathy from HCWs- challenged communication between HCWs and patient	▪ “At first, when I took those medications I was in boarding school. I was coughing all the time and children were laughing at me and I felt bad. I don’t know how the matron got to know but she knew and told them. They back-bitted [gossiped] me whenever I passed” [18 year-old female, Uganda] (Mutumba et al., 2015) ▪ “There is a girl we lost, she passed away, she was 18.. . she had [experienced] stigma at school because they came across her drugs in her suitcase, and they pulled them out and they put them there and put her [medical] card on her bed and she was a head-girl and that killed her [spirit]! She had to switch school. Most of them you get these calls, when they are saying they have found out, you see, so she had to switch out schools.” [Counsellor, Uganda] (Inzaule et al., 2016) ▪ “Sometimes when I don’t feel like taking my treatment, I don’t. I can’t take my pills with water, and if I don’t have juice, I simply can’t take them. (Matovu et al., 2012) They shout at us when we don’t take our treatment, just like they did today. I wouldn’t be able to say all these things I have said to you to anyone of them. They are strict with us, so we’re scared.” [20 year-old female, South Africa] (Goudge et al., 2009)
		- Sexual norms and gender oppression- impact on women’s mental health - HIV contraction via sexual intercourse- self-blame	▪ “They won’t understand that I got the HIV from my parents. They will think I was sleeping around with older men.” [(16 year-old female, South Africa] (Jena, 2014) ▪ “One of my older brothers once told us that if he heard that one of his sisters was HIV-positive, he’d kill her. I realised that my mother and my elder brother would never accept a person who was HIV-positive. That’s why I have decided to keep it to myself.” [20 year-old female, South Africa] (Goudge et al., 2009) ▪ “It’s my irresponsibility. I got infected through unprotected sexual intercourse. So, it’s irresponsibility.. .. No one deserves to get the virus. But when you didn’t care. .. sometimes I say I deserve it. I knew how to protect myself, I knew it. I was a promoter, a person who promoted condom use. But it happened, I don’t know how.” [22 year old female, South Africa] (Plattner and Meiring, 2006)
***1.2 Social support***	o Supportive and unsupportive networks and impact on mental health and wellbeing o Lack of support for caregivers o Longing for relationships	- Caregiver support-material support, treatment support, emotional support from parents, re-connecting with parents - Supportive siblings- forms of validation and acceptance - Supportive extended family- emotional support	▪ “I didn’t find any problem [with the drugs] because my mum used to encourage me to take it a lot. She was also on drugs so whenever she took hers, I also took mine” [15 year-old female, Uganda] (Mutumba et al., 2015) ▪ “At home they help me with everything and give me all the support I need. It helps me get through knowing they love me. We take our pills the same times so we always remind each other. When she takes hers, she calls me to take mine” (16 year-old female, South Africa) (Jena, 2014) ▪ “I believed that when one is positive he/she can die any time. I also felt am worthless in this world. Later my brothers came and assured me that there was no need of worrying much because they were there for me. They told me that they will take care of me.” [24 year-old female, Kenya] (Lypen et al., 2015) ▪ [My uncle] made that promise after my mother was buried; he told me — I’m going to support you in good and difficult times — and right now he still is.” [19 year-old, male, Botswana] (Midtbo et al., 2012)
		- Supportive peers- empathetic listening, encouragement - Supportive HCWs- gratitude for care, assisting with non-adherence, providing safe spaces for emotional release - HIV support group- received material support, instilled feelings of connectedness and acceptance	▪ “If I have stress, I can go to my friend’s place and explain to her and in turn she will give me advises [sic] that are worthy eventually the stress goes.” [19 year-old, male, Kenya] (Lypen et al., 2015) ▪ “I also didn’t accept myself, I cried and I was asking myself when I get to the house should I commit suicide or what? A nurse took me to a room and asked to cry until all the stress is gone. I really had stress.” [24 year-old female, Kenya] (Lypen et al., 2015) ▪ “Besides learning more about the disease, the pills and other things, they [who?] also provide me with money that I use to buy food...I feel welcomed. Like I have a family when I am with them. I always look forward to the meetings.” [15 year-old, male, eSwatini (formerly Swaziland)] (Shabalala et al., 2016) ▪ “I loved that children’s group because it comforted me to feel like I’m not the only one and to see that my friends have the same problem. […] And then we did not discriminate each other, we treated each other just like normal when we met. And we were not in a state of hatred and dislike but in a state of love,we loved each other just like normal.” [17 year-old female, Tanzania] (Mattes, 2014)
		- Unsupportive family networks- impact on coping, self-acceptance, social-acceptance - Lack of support for caregivers	▪ “My auntie told me that I do not belong to the family, because of my condition and I was always segregated from other family members. When I go back home my auntie starts throwing insults at me and saying that you have been sleeping around. She doesn’t care, if you tell her please aunt buy for me some clothes, she replies with annoyance that I stopped buying for you clothes in Primary five saying that I no longer have value and I don’t give you my things, its up to you. I remember the doctors called her one time to pick my medication and also to act as the adherence support person and she said, if it means for her to die, let it be so, I will not come. I even contemplated killing myself because of the situation. I secretly meet with my sisters, who financially support me and my auntie does not know, but when am in a hurry to meet with them I forget to take my medicine.” [16 year-old female, Uganda] (Matovu et al., 2012) ▪ “He did not see eye to eye with his sister-in-law who did not like the fact that Mpendulo was HIV positive...in one incidence the sister-in-law found him eating food from a plate that was not designated for him. She scolded the boy for using the plate; stating that...he will infect her children with HIV. That angered Mpendulo a lot. He said he felt unwelcomed and not wanted. “[Case study of 15 year-old male”, eSwatini (formerly Swaziland)] (Shabalala et al., 2016) ▪ “we just need a support group and I don’t know how it can be done. Some people believe they can just sit at home and cry which does not help, I know I have cried and I am still crying and have not found help yet.” [Caregiver, South Africa] (Petersen et al., 2010)
		- Multiple losses and complicated grieving- longing for relationships they never got to experience - Longing for fathers- shaped by deep cultural expectations	▪ “It’s that every child wants to have a dad and a mother at the same time … growing up having a dad and a mother because it’s really sad seeing some of my friends having their families and telling me they went out with their dads, then I knew that I didn’t have a dad … so many children do suffer from that thing because you all want parents, both parents.” [17 year-old male, South Africa] (Woollett et al., 2017) ▪ “I sometimes feel like it is empty here [pointing on the left side of his chest], like there is this big hole...like if I had a relationship with my real father, singavaleka lesikhala lengisivako la [this hole I feel in here would be closed]” [15 year-old, male, eSwatini (formerly Swaziland)] (Shabalala et al., 2016)
**Theme 2: Coping**			
	o Positive coping strategies facilitates wellbeing o Negative coping strategies and impact on mental health	- Religion and faith- draw on beliefs and values to cope with stressful situation, relationship with God, source of comfort and hope, brings a sense of meaning and purpose	▪ “So you know they say God throws challenges at you to make you stronger; God does not throw things that He knows that you cannot handle? He throws things at you that He knows that you can handle...so that’s what keeps me going and to me like that’s what tells me everything happens for a reason. There is a reason it happened [becoming HIV positive] and cannot be changed now and if I want to continue to live, I have to take my tablets and all that...so that’s what keeps me going” [18 year-old male, South Africa] (Woollett et al., 2016) ▪ “God is going to give me all of my wishes, my dreams. He’s going to. .. God will be there” [15 year-old, male, South Africa] (Li et al., 2010) ▪ “I have accepted the Lord. I don’t know but if I were not [HIV-] positive, perhaps I would not have accepted the Lord. But it is being positive that makes you turn back from the world so you could also think about God” [24 year-old, female, Namibia] (Plattner and Meiring, 2006)
		- Aspirations- marriage, children, educational attainment, career goals	▪ “I definitely want to be married and have my own family and children too when I finish my studies” [16 year-old female, South Africa] (Jena, 2014) ▪ “I want to be someone in future, a person that people admire and respects and going to school is my stepping stone” [16 year-old female, South Africa] (Jena, 2014) ▪ “I want to be a medical doctor and I want to study medicine. Am in Science class. So this picture reminds me of it that I can achieve that goal” [17 year-old, female, Nigeria] (Adegoke and Steyn, 2017)
		- Normalising one’s HIV condition- self-acceptance, not feeling alone, social comparisons	▪ “You are just like a normal person, that means you live, you do your business, you study, you finish, you find work, you can support yourself. So to have [HIV] is like having a common fever.” [19 year-old male, Tanzania] (Mattes, 2014) ▪ “When I am dancing, even being with HIV, I am as normal as other children.” [15–19 year-old, male, South Africa] (Rosenbaum, 2017) ▪ “I am happy with it because there are some diseases which are bigger than this disease like cancer.” [18 year-old female, Uganda] (Mutumba et al., 2015)
		- Social isolation - Blame - Anticipation of fearful events	▪ “He is always lonely and unhappy until sometimes I cheat him [I tell him] that do not worry you no longer have the virus ...” [Grandmother-caregiver, Kenya] (Abubakar et al., 2016) ▪ “They are always asking “why me, why me?” and sometimes they blame and resent their parents.” [Caregiver, Uganda] (Loos et al., 2013) ▪ “Yeah and afterwards, after like 3 years my mom died. I was like “I’m the next, I’m the next, I’m the chosen one”. Then my uncle dies and I was like “shit” … this shit is a really huge measure thing. Over fast, like you’re going down …;. I don’t know, this thing keep on telling me that [I will die], I don’t know why, so yeah … Yeah, it’s like they are beating me up with a five pound hammer, you see, shot after shot, shot after shot, so yeah.” [18 year-old, male, South Africa] (Woollett et al., 2017)
**Theme 3- Standard of living**			
***Economic insecurity***	o Food insecurity and impact on adherence and mental health o Fulfilling socio-cultural roles important for wellbeing	- Hunger and adherence	▪ “The main challenge, they are complaining a lot about hunger. They say because of medication they need a lot of food and you see most of their guardians are not financially able...” [Community healthcare worker, female, Kenya] (Abubakar et al., 2016) ▪ “It is because (crying) I sometimes get short of the money. .. it is sometimes so difficult for me to come and collect her medication because of the lack of money. .. I am unable to buy the right food for her because she has a special diet since she is sick” [Caregiver, South Africa] (Petersen et al., 2010)
		- Limited schooling- unable to engage in occupations they aspired to - Economic scarcity- delay on sexual debut, marriage, having children, limits ability to feel socially valued	▪ “I really wanted to be a teacher. I was not able to realize this goal. I did not have enough financial ability to help me pursue this goal. ...My parents died long ago. I had to come back from school every evening and look for money, at times I had to miss school because I had no pens.” …. [24-year old male, Uganda] (Mathur et al., 2016) ▪ “Until I have built a house for myself, when I have a house like this [referring to his mother’s house] I can slowly start thinking about getting married. But […] for example if you fail Form IV, you get married, do you have a house to put your girl in? Do you have work to feed your child? You have to fight to get a good job, to build a house, to prepare well. […] Right now […] I’m concentrating on books [education], that’s it!” [16 year-old male, Tanzania] (Mattes, 2014) ▪ “But he would feel hurt when his uncle complained that Mpendulo did not contribute to the household. He felt his inability to contribute was caused by his brother’s refusal to process his share of their father’s estate, and he himself carried the brunt of this as lack of money often forced him to take his medication on an empty stomach.” [15 year-old, male, eSwatini (formerly Swaziland)] (Shabalala et al., 2016)

All the studies highlighted the role HIV-related stigma and discrimination played in reinforcing social isolation and poor mental health functioning among YPLHIV. Perceived contributors included forms of internalised stigma, characterised as fear of the rejection and isolation if HIV status was accidentally disclosed to peers [100], partners [101–104] or parents [103, 105, 106]. Hence, keeping one’s HIV status undisclosed was often described as an everyday lived experience [107–110]. Studies described how internalised stigma impeded ability to build “healthy identities” [104, 108, 111] and access social support [101]. Gendered social expectations appeared to facilitate internalised stigma and consequently limited disclosure. For example, in Uganda, a young woman living with HIV feared loss of social value if her intimate relationship was curtailed and hence opted for non-disclosure of her HIV status [101]. Similarly, young men living with HIV in Uganda felt unable to uphold masculine socio-cultural identities and thus chose not to disclose their HIV status to their parents in fear of the “loss of rights” to endowments [105, 106]. Most studies described the manifestations of HIV-related stigma in terms of experienced stigma, which was encountered in relationships with mothers [112, 113], family members [101, 114], learners and educators (102Mutumba et al., 2015, 107, 115), and HCWs [115] [113]. Authors also described how stigma intersected with gendered attitudes towards sexuality and self-acceptance among young women [101, 115–117], including cultural norms such as respect for elders [113, 118, 119].

b) ***Social support***

The importance of relationships and social connections was expressed in several studies. Shabalala, De Lannoy [114] notes that for YPLHIV in eSwatini (formerly called Swaziland) strong relationships with caregivers were linked with “being accepted, being connected and welcomed”. Mutumba, Bauermeister [102] describes how pill taking between HIV-positive parents and YPLHIV in Uganda fostered bonding “whenever she took hers, I also took mine”. In contrast, in three studies, young women expressed the lack of support from caregivers [101, 112, 120]. Trusting relationships with family members were frequently described as important [101, 102, 109, 121, 122]. In a Kenyan study, authors indicate that the acceptance received from siblings served to “validate the participant as a human being” [109]. Unsupportive family networks were also described in the literature in the context of poor mental health functioning and reduced wellbeing (i.e. lack of self-acceptance) [101, 114].

Furthermore, studies described how YPLHIV drew on their friendship networks to manage treatment adherence, seek advice and feel accepted [104, 109, 123]. Several participants expressed gratitude for the informational and emotional support received from HCWs [107, 109, 119, 124]. Furthermore, in settings were YPLHIV had access to support groups, feeling “comforted” [103], “normal” [103] “welcomed” [114], “open to share” [121], and “not alone” [111] were dominant in the narratives. Caregivers in a South African study highlighted their lack of social support and how it hindered their ability to cope and subsequently support their children [104]. In three studies, YPLHIV articulated a deep longing for their deceased parents which authors indicated signified the importance parents played in nurturing belonging and its link with creating meaning in life [104, 114, 117, 120]. Moreover, the yearning for one’s father was interpreted as being underpinned by “cultural expectations of being cared for and finding a rightful place in their father’s home” [114].

ii. **Coping**

A key positive factor that YPLHIV utilised for coping was religion and faith, especially when support was limited. Religion and faith were primarily expressed as “belief in God” [100, 109, 111, 112, 116, 121, 123, 125]. Most authors perceived religion as bringing comfort and hope, as well as meaning and purpose in their lives. In addition, in a Namibian study, interpreting HIV acquisition as a “test or punishments from God” helped a young woman to accept her HIV status [116]. Another major positive coping strategy that YPLHIV applied was future goal setting such as desire for marriage and children [107, 119, 125, 126]. Educational aspirations were perceived as bringing a sense of purpose or social value to participants’ lives [107, 122]. In contrast, YPLHIV also utilised negative coping strategies such as social withdrawal [107, 108, 111], self-blame [102, 123] and anticipation of death [103, 104, 117].

iii. **Standard of living**

Most YPLHIV resided in non-nuclear and skipped-generation households that had encountered multiple losses. These households were often described by authors as lacking economic security and social protection. In Kenya, Zimbabwe and South Africa, caregivers and community HCWs reported how food insecurity challenged ART adherence and positive mental health functioning among YPLHIV [104, 110, 111, 127]. Young men living with HIV often reported difficulty in establishing economic security as the sole reason for delaying sexual debut, marriage and having children [100, 103, 126]. In Uganda, two studies noted how young men had “shattered dreams” [112] as household poverty traps prevented young men from completing their schooling, achieving their career aspirations and entering into the formal labour market. A young man’s inability to contribute to his household made him feel “unwanted” and “hurt” [114].

#### Quality of studies

Twenty-seven out of 30 qualitative studies were of medium (*n* = 19) to high quality (*n* = 8) (Table 7). The remaining three studies were of low quality largely due to insufficient information on the methods and participant sample, including lack of description in the analysis. For most studies included in this synthesis, findings were presented clearly with concrete detail and discussed in relation to other literature and theories.

**Table 7:**
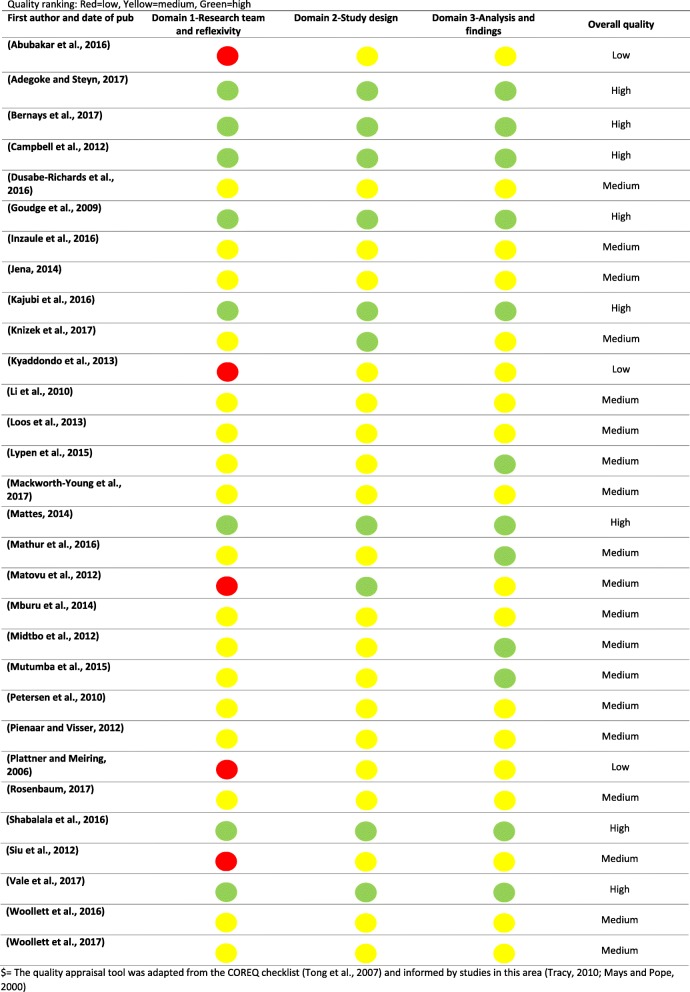
Quality appraisal ^$^ of full-text studies included in the qualitative synthesis (*N* = 30). These studies examined lived experiences related to dimensions of wellbeing or mental health among YPLHIV in SSA

$ = The quality appraisal tool was adapted from the COREQ checklist (Tong et al., 2007) and informed by studies in this area (Tracy, 2010; Mays and Pope, 2000).

Based on the CERQual assessment, we report moderate confidence in most themes (Table 8).

**Table 8:** Assessment of the quality of the qualitative evidence using the CERQual approach. This evidence examined themes that shaped experiences suggestive of wellbeing among YPLHIV in SSA

Summary of review finding	Studies contributing to review findings	Methodological limitations	Coherence	Adequacy	Relevance	CERQual assessment of confidence in the evidence	Explanation of CERQual assessment
**Theme 1-Social acceptance and belonging**							
1.1. ***HIV-related stigma and discrimination*** Stigma negatively impacted wellbeing by reducing self-acceptance and challenging the ability to maintain positive relationships. Internalised and externalised stigma impeded the ability to derive meaning in and to life and consequently wellbeing. It negatively impacted self-worth, connectedness with others and self-acceptance, particularly among women. Moreover, it challenged the ability to build relationships and reciprocate love and affection.	30	Minor (16 studies with minor and 11 studies with moderate methodological concerns- i.e. methodological orientation, reflexivity)	Minor (Few concerns on the data from the primary data and review finding)	Minor (Most studies provided thick and rich descriptions on this theme)	Moderate (No study was informed by or discussed in the context of wellbeing theory, geographical spread- 15 studies from eastern sub-region, 15 studies from the southern sub-region, 1 study from West Africa, most studies conducted among 15–19 year-olds living with HIV	Moderate confidence	Minor concerns regarding methodological limitations, coherence and adequacy Moderate concerns regarding relevance
1.2 ***Social support*** Positive relations were critical in promoting wellbeing. Supportive relationships with caregivers and trusting relationships with extended family members, peers, HCWs and support groups enhanced social acceptance and belonging. This in turn promoted a sense of meaning in and to life, and ultimately wellbeing Unsupportive relationships reinforced feelings of social isolation. Moreover, sexual norms embedded within these networks compromised wellbeing, particularly among women.	28	Minor (15 studies with minor and 11 studies with moderate methodological concerns- i.e., methodological orientation, reflexivity)	Minor (Few concerns on the data from the primary data and review finding)	Minor (Most studies provided thick and rich descriptions on this theme)	Moderate (No study was informed by or discussed in the context of wellbeing theory, geographical spread- 14 studies conducted in the eastern sub-region, 14 studies conducted in the southern sub-region, 1 study from West Africa, most studies conducted among 15–19 year-olds living with HIV	Moderate confidence	Minor concerns regarding methodological limitations, coherence and adequacy Moderate concerns regarding relevance
**Theme 2- Coping**							
The ability to manage daily lived realities was important for wellbeing. YPLHIV drew on religion and faith to help understand the meaning of life. This may have engendered a sense of control, belongingness and relatedness and thereby brought meaning to and in life. Similarly, goals and aspirations brought meaning and purpose to life. Strong social support networks fostered positive coping. Negative coping strategies such as social withdrawal, self-blame and anticipation of death reduced ability to finding meaning in life and thus undermined wellbeing	23	Minor (5 studies with minor and 18 studies with moderate methodological concerns- i.e. methodological orientation, reflexivity)	Minor (Few concerns on the data from the primary data and review finding)	Minor (Most studies provided thick and rich descriptions on this theme)	Moderate (No study was informed by or discussed in the context of wellbeing theory, geographical spread- 12 studies from eastern sub-region, 11 studies from the southern sub-region, 1 study from West Africa, most studies conducted among 15–19 year-olds living with HIV	Moderate confidence	Minor concerns regarding methodological limitations, coherence and adequacy Moderate concerns regarding relevance
**Standard of living**							
Fulfilling socio-economic roles were important for wellbeing. It served to enhance meaning in and to life and created purpose in life. Household food insecurity compromised ART adherence and positive mental health functioning and possibly wellbeing. Broader economic constraints challenged the ability of young men to attain desired educational and career goals. This reduced their sense of social value and threatened wellbeing.	11	Moderate (4 studies with minor and 7 studies with moderate methodological concerns- i.e. reflexivity, lack of thick descriptions in the analysis or description of diverse cases)	Moderate (Several concerns on the data from the primary data and review finding)	Moderate (Few studies provided thick and rich descriptions on this theme)	Moderate (No study was informed by or discussed in the context of wellbeing theory, majority of the data are from men and caregivers. Geographical spread- 5 studies conducted in southern sub-region, 5 studies conducted in the eastern sub-region, 1 study conducted in western sub-region	Moderate confidence	Moderate concerns regarding methodological limitations, coherence, adequacy, relevance

### Overall synthesis

Both our quantitative and qualitative evidence suggest that social networks were at the core of wellbeing for YPLHIV, which supports a relational construct of wellbeing among YPLHIV. Drawing on the relationality meaning model [60], our findings suggest that social relationships were critical in fostering belongingness and connectedness. This in turn contributed to a sense of meaning in and to life, and ultimately wellbeing. In addition, socio-cultural norms and values were important to the wellbeing of YPLHIV, as these helped realise the meaning of and in life. In contrast, our synthesis also revealed that in certain instances, social networks and gender norms embedded within these networks compromised wellbeing. Based on our evidence, dimensions that potentially constitute wellbeing for YPLHIV can be mapped onto the following PWB dimensions: 1) self-acceptance — internalised stigma, externalised stigma, social acceptance; 2) belonging — family connectedness; 3) autonomy — disclosure, sexual intercourse; 4) positive relations — social support; 5) environmental mastery — positive and negative coping; 6) purpose in life — religious activities, educational aspirations .

## Discussion

Through this mixed method review we sought to identify key dimensions of wellbeing among YPLHIV in SSA by synthesising the evidence on correlates and experiences of wellbeing. The goal of this review was to inform wellbeing measurement selection for health economic surveys that seek to evaluate the impact of HIV policies on wellbeing. Findings from our review indicate that key dimensions which define wellbeing for this population include social support, family belonging, self-acceptance, coping and purpose. These results suggest that multi-dimensional wellbeing measures with a strong focus on social relationships may be appropriate for this setting.

Economic studies from high income settings have found robust positive associations between social ties (e.g. family, peers, neighbours) and SWB (life satisfaction, happiness) [128]. However, our findings suggest that social networks have both benefits and liabilities for wellbeing among YPLHIV in this setting, which is consistent with recent evidence that highlights the positive and negative relational mechanisms of social networks on SWB [129], particularly among adolescents [130]. Our quantitative synthesis showed a strong positive correlation between HIV-related stigma and poor mental functioning which may likely reduce overall wellbeing as shown in previous studies among adults living with HIV in SSA [131] and high income countries [132]. Drawing on our qualitative review findings together with the relational meaning model [60] and Goffman’s accounts of stigma [133], internalised stigma may reduce wellbeing by lowering self-worth. In addition, externalised stigma encountered within social networks may reduce connectedness of self with family members and peers [102, 112, 134]. Together, these processes are likely to challenge self-acceptance. The effects of stigma on wellbeing are likely be more pronounced for sexually HIV-positive young women, who, due to the intersection of stigma with sexual norms, may harbour heightened feelings of shame and rejection [113, 116]. These findings suggest that wellbeing measures which focus on self-acceptance and self-worth may be appropriate for this population.

Our synthesis also highlighted the importance of social relationships in bringing meaning to life for YPLHIV. However, caregivers often limited the decision-making of YPLHIV with regards to disclosure [102] and sexual intercourse [119], which may have reduced the ability of YPHIV to build relationships and reciprocate affection. In addition, the absence of relationships, particularly with fathers, may have reduced belongingness [114]. Family belonging has been shown to mediate pathways to SWB [135] and PWB [136] among adolescents in developed settings, possibly via promotion of meaning in life [137]. Overall, these findings highlight the relevance of selecting wellbeing measures with dimensions designed to capture family belongingness and autonomy.

Our review revealed a link between strong social support networks and positive mental health among YPLHIV. It is plausible that these strong social networks also promote PWB as shown in a previous adolescent study from Brazil [138]. Accounts from our qualitative synthesis suggest that YPLHIV drew on the emotional support from family, peers and HCWs members during challenging life events (i.e. disclosure, adherence challenges, rejection) which may have helped maintain positive mental health and potentially PWB [102, 103, 109]. However, in instances where supportive social networks were limited, we found that YPLHIV drew on negative coping mechanisms (e.g. social withdrawal, self-blame) [104], which likely reduced their PWB [139]. Our findings suggest that social support positively impacts PWB by adding meaning to life, specifically on an intra-personal level such as improving one’s sense of environmental mastery (e.g. ART adherence), a dimension of PWB. Our findings suggest that wellbeing measures should also encompass dimensions related to positive relations and environmental mastery.

Our review showed that socio-cultural values, norms and beliefs were critical for creating purpose in life among YPLHIV, which is also considered important for positive youth development [140]. Results from our qualitative synthesis suggest that religious beliefs, values and practices, helped YPLHIV understand the meaning of life, particularly in relation to their HIV status [116], which is agreement with previous adolescent studies [100, 125, 141, 142]. Educational and employment aspirations were commonly reported in our qualitative evidence [107, 122, 126], and may have helped promote wellbeing by creating a sense of purpose in life [43]. In addition, the desire for economic security among males [103, 114, 126] also suggests that their wellbeing may be rooted in their ability to fulfil socio-cultural roles such as breadwinners, and thereby add purpose to their lives. These findings suggest that wellbeing measures that consider domains such religion living standards or dimensions such as purpose in life might be valuable for this population.

Overall, our findings support the use of multi-dimensional relational wellbeing scales aligned to life domains which are important to YPLHIV (i.e. family, religion, education, living standards). Subjective wellbeing scales that consider these domains include the Personal Wellbeing Index [42] and the Student Life Satisfaction Scale [41]. The Personal Wellbeing Index has been applied in South Africa’s national social attitudes survey of individuals aged 16 years and older [143]. The Personal Wellbeing Index has exhibited good scale reliability among adolescents in developing countries [144] whereas the Student Life Satisfaction Scale has shown favourable psychometric properties among children in South Africa [145]. Broader wellbeing scales such as Ryff’s PWB scale and the Mental Health Continuum Short- Form have shown strong alignment with themes that emerged in our synthesis (i.e. social support, belonging, purpose in life, self-acceptance). These PWB scales have shown good validity among adolescent populations in the North [146]. In addition, the Mental Health Continuum Short- Form has shown strong alignment with conceptualisation of a good life among adolescents in South Africa [65]. Further studies are needed to validate these wellbeing measures among YPLHIV in SSA, paying careful attention to translational issues [147, 148]. These proposed scales are relatively brief and can be either self- or interviewer- administered in national economic or HIV surveys, including programme evaluations. In addition, key dimensions of wellbeing in this setting (e.g. positive relations, acceptance, coping) could be used as key output measures in monitoring and evaluation frameworks of multi-sectoral HIV policies and programmes to indirectly assess wellbeing impacts. Our findings also highlight the importance of including social support, stigma, gender and living standards as explanatory variables in econometric models examining the wellbeing effects of HIV/AIDS policies among YPLHIV.

Key strengths of this review include: 1) use of mixed-methods, with qualitative data used to explain patterns in the quantitative synthesis; 2) use of a search strategy with a comprehensive definition of wellbeing that allowed us to examine wellbeing literature from multiple disciplines; 3) inclusion of both peer-reviewed articles and grey literature; 4) quality appraisal of each included study and quality assessment of the synthesised evidence.

This review is subject to the following limitations: 1) key population groups such as LGTBQI, sex workers and injecting drug users were excluded, as their lived experiences are likely to be different given the higher levels of stigma they possibly encounter relative to the general population of YPLHIV [149–151]; 2) several applicable regional conferences lack online abstract and thus our review may have missed potentially relevant material; 3) our review focused only on YPLHIV in SSA and thus findings may not be generalisable to other regions. However, results from recent studies suggest that there are similarities between YPLHIV in SSA and other developing nations within the South-East Asia Region and Region of the Americas in terms of correlates of mental illness [81, 152] and adherence [153, 154]. Hence, results could be relevant to settings with similar disease and socio-economic profiles. Several gaps in our evidence base were identified. First, no study directly assessed correlates of wellbeing (using a wellbeing scale) or evaluated experiences of subjective or psychological wellbeing. Second, there were no data from Central Africa, with only one study from West Africa. Third, our evidence is not generalisable to YPLHIV in the community given that most studies sampled individuals from healthcare facilities. Fourth, variability in outcome definition and scale choice made it difficult to compare between quantitative studies and did not allow for a meta-analysis. Fifth, the lack of age-stratified data limited our ability to compare correlates and wellbeing by relevant age-bands (15–19 vs. 20–24 yrs.). Sixth, weaknesses in the study design and analytical techniques used in the quantitative evidence limited out ability to draw out any causal interpretations. Lastly, for the qualitative review, data saturation among 20–24-year-olds and YPLHIV in rural settings was not reached, leaving gaps in our understanding of how these factors may have shaped wellbeing.

## Conclusion

The aim of this mixed-methods review was to identify dimensions of wellbeing among YPLHIV and measures that align to these dimensions for application in HIV policy evaluations. Understanding the wellbeing effects of HIV/AIDS policies could help steer policies in the direction that meets the broader needs of YPLHIV. This review has shown that social support was a key correlate of poor mental health and that social relationships shaped positive lived experiences. In view of the negative association between poor mental health functioning and measures of wellbeing found in the literature, it is plausible to posit that in this population wellbeing is multi-dimensional and that relational dimensions frame wellbeing among YPLHIV. Multi-dimensional wellbeing scales with a strong relational focus that could be applicable for this group include the Personal Wellbeing Index, Ryff’s PWB and Mental Health Continuum Short-Form. Future studies should go beyond the investigation of mental health and examine wellbeing, based on definitions grounded in theory, to provide more accurate data on the wellbeing effects of policies. However, psychometric evaluations of these scales in this population in SSA are warranted, together with validation of these scales against adolescents and young people’s subjective experiences in SSA.

## Supplementary information

**Additional file 1.** PRISMA Checklist.

**Additional file 2.** ENTREQ Checklist.

**Additional file 3.** Search strategy-MEDLINE (OVID).

**Additional file 4.** Search strategy- PsychInfo (OVID).

**Additional file 5.** Search strategy- Econlit (OVID).

**Additional file 6.** Search strategy- AfricaWide (EMBASE).

**Additional file 7.** Search strategy- Web of Science.

**Additional file 8.** Search strategy- ProQuest.

**Additional file 9.** Search strategy- IAS conference abstract archive.

**Additional file 10.** Search strategy- Other databases.

**Additional file 11. Table 4.** Quantitative review outcomes- additional.

## Data Availability

The datasets used in the current study is available from the corresponding author on request.

## Abbreviations

AIDS: Acquired immune deficiency syndrome
ART: Antiretroviral therapy
HIV: Human immunodeficiency virus
LGTBQI: Lesbian, gay, bisexual, transgender, transsexual, queer, questioning, intersex,
PWB: Psychological wellbeing
SDG: Sustainable Development Goals
SSA: Sub-Saharan Africa
SWB: Subjective wellbeing
WHO: World Health Organization
YPLHIV: Young people living with HIV
