# Supplementary material for: Informing the measurement of wellbeing among young people living with HIV in sub-Saharan Africa for policy evaluations: a mixed-methods systematic review
Source: Health Qual Life Outcomes. 2020 May 5;18:120. doi: 10.1186/s12955-020-01352-w (PMC7201613; doi:10.1186/s12955-020-01352-w)
Supplement: Supplementary file 2 — Additional file 2. ENTREQ Checklist. [file 12955_2020_1352_MOESM2_ESM.docx]

Additional file 2: ENTREQ Statement

| **No Item Guide and description** | **No Item Guide and description** | **No Item Guide and description** | **Page** |
| --- | --- | --- | --- |
| 1 | Aim | State the research question the synthesis  addresses. | 4-5 |
| 2 | Synthesis  methodology | Identify the synthesis methodology or  theoretical framework which underpins  the synthesis, and describe the rationale  for choice of methodology *(e.g. metaethnography,*  *thematic synthesis, critical*  *interpretive synthesis, grounded theory*  *synthesis, realist synthesis, metaaggregation,*  *meta-study, framework*  *synthesis)* | 6-7, 12-13 |
| 3 | Approach to  searching | Indicate whether the search was preplanned  (*comprehensive search strategies*  *to seek all available studies)* or iterative  (*to seek all available concepts until they*  *theoretical saturation is achieved)*. | 10 |
| 4 | Inclusion criteria | Specify the inclusion/exclusion criteria  (e.g. in terms of population, language,  year limits, type of publication, study  type). | 8-10, Table 1 |
| 5 | Data sources | Describe the information sources used  (e.g. electronic databases (MEDLINE,  EMBASE, CINAHL, psycINFO, Econlit), grey  literature databases (digital thesis, policy  reports), relevant organisational websites, *experts, information specialists, generic*  *web searches (Google Scholar) hand*  *searching, reference lists)* and when the  searches conducted; provide the rationale  for using the data sources. | 10, Appendix 3-10 |
| 6 | Electronic Search  strategy | Describe the literature search *(e.g. provide*  *electronic search strategies with*  *population terms, clinical or health topic*  *terms, experiential or social phenomena*  *related terms, filters for qualitative*  *research, and search limits)*. | 10, Appendix 3-10 |
| 7 | Study screening  methods | Describe the process of study screening  and sifting (e.g. title, abstract and full text  review, number of independent reviewers  who screened studies). | 10-11 |
| 8 | Study  characteristics | Present the characteristics of the included  studies (e.g. year of publication, country,  population, number of participants, data  collection, methodology, analysis, research  questions). | 14, 19, Table 3 |
| 9 | Study selection  results | Identify the number of studies screened  and provide reasons for study exclusion  (e,g, for comprehensive searching, provide  numbers of studies screened and reasons  for exclusion indicated in a  figure/flowchart; for iterative searching  describe reasons for study exclusion and  inclusion based on modifications t the  research question and/or contribution to  theory development). | 14, 19, Figure 1 |
| 10 | Rationale for  appraisal | Describe the rationale and approach used  to appraise the included studies or  selected findings (e.g. assessment of  conduct (validity and robustness),  assessment of reporting (transparency),  assessment of content and utility of the  findings). | 12 |
| 11 | Appraisal items | State the tools, frameworks and criteria  used to appraise the studies or selected  findings (e.g. Existing tools: CASP, QARI,  COREQ, Mays and Pope; reviewer  developed tools; describe the domains  assessed: research team, study design,  data analysis and interpretations,  reporting). | 12 |
| 12 | Appraisal  process | Indicate whether the appraisal was  conducted independently by more than  one reviewer and if consensus was  required. | 12 |
| 13 | Appraisal results | Present results of the quality assessment  and indicate which articles, if any, were  weighted/excluded based on the  assessment and give the rationale. | 23, Table 7 |
| 14 | Data extraction | Indicate which sections of the primary  studies were analysed and how were the  data extracted from the primary studies?  (e.g. all text under the headings “results  /conclusions” were extracted electronically  and entered into a computer software). | 10-11 |
| 15 | Software | State the computer software used, if any. | 10 |
| 16 | Number of  reviewers | Identify who was involved in coding and  analysis. | 10, 11-12 |
| 17 | Coding | Describe the process for coding of data  (e.g. line by line coding to search for  concepts). | 13 |
| 18 | Study  comparison | Describe how were comparisons made  within and across studies *(e.g. subsequent*  *studies were coded into pre-existing*  *concepts, and new concepts were created*  *when deemed necessary).* | 12-13 |
| 19 | Derivation of  themes | Explain whether the process of deriving  the themes or constructs was inductive or  deductive. | 12-13 |
| 20 | Quotations | Provide quotations from the primary  studies to illustrate themes/constructs,  and identify whether the quotations were  participant quotations of the author’s  interpretation. | Table 6 |
| 21 | Synthesis output | Present rich, compelling and useful results  that go beyond a summary of the primary  studies (e.g. *new interpretation, models of*  *evidence, conceptual models, analytical*  *framework, development of a new theory*  *or construct).* | 22-23, Table 8 |
