# Supplementary material for: Informing the measurement of wellbeing among young people living with HIV in sub-Saharan Africa for policy evaluations: a mixed-methods systematic review
Source: Health Qual Life Outcomes. 2020 May 5;18:120. doi: 10.1186/s12955-020-01352-w (PMC7201613; doi:10.1186/s12955-020-01352-w)
Supplement: Supplementary file 3 — Additional file 3. Search strategy-MEDLINE (OVID). [file 12955_2020_1352_MOESM3_ESM.docx]

Additional file 3: Ovid Medline search strategy

| 1. Quality of Life.mp. or exp "Quality of Life"/ |
| --- |
| 2. health-related quality of life.mp. |
| 3. wellbeing.mp. |
| 4. well-being.mp. |
| 5. (subjective adj1 wellbeing).mp. [mp=title, abstract, heading word, table of contents, key concepts, original title, tests & measures] |
| 6. (psychological adj1 wellbeing).mp. [mp=title, abstract, heading word, table of contents, key concepts, original title, tests & measures] |
| 7. (life adj1 satisfaction).mp. [mp=title, abstract, heading word, table of contents, key concepts, original title, tests & measures] |
| 8. (health adj1 satisfaction).mp. [mp=title, abstract, heading word, table of contents, key concepts, original title, tests & measures] |
| 9. (well adj1 being).mp. |
| 10. exp Personal Satisfaction/ |
| 11. exp Emotions/ |
| 12. exp Depression/ec, ep, px [Economics, Epidemiology, Psychology] |
| 13. exp Mental Health/ |
| 14. exp Personal Autonomy/ |
| 15. exp Mental Competency/ |
| 16. exp Resilience, Psychological/ |
| 17. exp Social Support/ or exp Social Capital/ |
| 18. attitude/ or optimism/ or pessimism/ |
| 19. (subjective adj1 well-being).mp. [mp=title, abstract, heading word, table of contents, key concepts, original title, tests & measures] |
| 20. (psychological adj1 well-being).mp. [mp=title, abstract, heading word, table of contents, key concepts, original title, tests & measures] |
| 21. mood.mp. |
| 22. Attitude/ |
| 23. Sociological factors/ |
| 24. Self-concept/ |
| 25. (relational adj1 well-being).mp. [mp=title, abstract, heading word, table of contents, key concepts, original title, tests & measures] |
| 26. (relational adj1 wellbeing).mp. [mp=title, abstract, heading word, table of contents, key concepts, original title, tests & measures] |
| 27. (lived adj1 experience$).mp. [mp=title, abstract, heading word, table of contents, key concepts, original title, tests & measures] |
| 28. happiness.mp. or HAPPINESS/ |
| 29. (psychological adj1 functioning).mp. [mp=title, abstract, heading word, table of contents, key concepts, original title, tests & measures] |
| 30. or/1-29 |
| 31. Benin/ |
| 32. (Benin or Dahomey).mp. [mp=title, abstract, heading word, table of contents, key concepts, original title, tests & measures] |
| 33. Burkina Faso/ |
| 34. (Burkina Faso or Burkina Fasso or Upper Volta).mp. [mp=title, abstract, heading word, table of contents, key concepts, original title, tests & measures] |
| 35. Burundi/ |
| 36. Burundi.mp. [mp=title, abstract, heading word, table of contents, key concepts, original title, tests & measures] |
| 37. Central African Republic/ |
| 38. (Central African Republic or Ubangi-Shari).mp. [mp=title, abstract, heading word, table of contents, key concepts, original title, tests & measures] |
| 39. Chad/ |
| 40. Chad.mp. [mp=title, abstract, heading word, table of contents, key concepts, original title, tests & measures] |
| 41. Comoros/ |
| 42. (Comoros or Comoro Islands or Mayotte or Iles Comores).mp. [mp=title, abstract, heading word, table of contents, key concepts, original title, tests & measures] |
| 43. "Democratic Republic of the Congo"/ |
| 44. ((democratic republic adj2 congo) or belgian congo or zaire).mp. [mp=title, abstract, heading word, table of contents, key concepts, original title, tests & measures] |
| 45. Eritrea/ |
| 46. Eritrea.mp. [mp=title, abstract, heading word, table of contents, key concepts, original title, tests & measures] |
| 47. Ethiopia/ |
| 48. Ethiopia.mp. [mp=title, abstract, heading word, table of contents, key concepts, original title, tests & measures] |
| 49. Gambia/ |
| 50. Gambia.mp. [mp=title, abstract, heading word, table of contents, key concepts, original title, tests & measures] |
| 51. Guinea/ |
| 52. (Guinea not (New Guinea or Guinea Pig* or Guinea Fowl)).mp. [mp=title, abstract, heading word, table of contents, key concepts, original title, tests & measures] |
| 53. Guinea-Bissau/ |
| 54. (Guinea-Bissau or Portuguese Guinea).mp. [mp=title, abstract, heading word, table of contents, key concepts, original title, tests & measures] |
| 55. Liberia/ |
| 56. Liberia.mp. [mp=title, abstract, heading word, table of contents, key concepts, original title, tests & measures] |
| 57. Madagascar/ |
| 58. (Madagascar or Malagasy Republic).mp. [mp=title, abstract, heading word, table of contents, key concepts, original title, tests & measures] |
| 59. Malawi/ |
| 60. (Malawi or Nyasaland).mp. [mp=title, abstract, heading word, table of contents, key concepts, original title, tests & measures] |
| 61. Mali/ |
| 62. Mali.mp. [mp=title, abstract, heading word, table of contents, key concepts, original title, tests & measures] |
| 63. Mozambique/ |
| 64. (Mozambique or Portuguese East Africa).mp. [mp=title, abstract, heading word, table of contents, key concepts, original title, tests & measures] |
| 65. Niger/ |
| 66. (Niger not (Aspergillus or Peptococcus or Schizothorax or Cruciferae or Gobius or Lasius or Agelastes or Melanosuchus or radish or Parastromateus or Orius or Apergillus or Parastromateus or Stomoxys)).mp. [mp=title, abstract, heading word, table of contents, key concepts, original title, tests & measures] |
| 67. Rwanda/ |
| 68. (Rwanda or Ruanda).mp. [mp=title, abstract, heading word, table of contents, key concepts, original title, tests & measures] |
| 69. Senegal/ |
| 70. senegal.mp. |
| 71. Sierra Leone/ |
| 72. Sierra Leone.mp. |
| 73. Somalia/ |
| 74. Somalia.mp. [mp=title, abstract, heading word, table of contents, key concepts, original title, tests & measures] |
| 75. South Sudan/ |
| 76. south sudan.mp. |
| 77. Tanzania/ |
| 78. (Tanzania or Zanzibar).mp. [mp=title, abstract, heading word, table of contents, key concepts, original title, tests & measures] |
| 79. Togo/ |
| 80. (Togo or Togolese Republic).mp. [mp=title, abstract, heading word, table of contents, key concepts, original title, tests & measures] |
| 81. Uganda/ |
| 82. Uganda.mp. [mp=title, abstract, heading word, table of contents, key concepts, original title, tests & measures] |
| 83. Zimbabwe/ |
| 84. (Zimbabwe or Rhodesia).mp. [mp=title, abstract, heading word, table of contents, key concepts, original title, tests & measures] |
| 85. Cameroon/ |
| 86. Cameroon.mp. [mp=title, abstract, heading word, table of contents, key concepts, original title, tests & measures] |
| 87. Cape Verde/ |
| 88. (Cape Verde or Cabo Verde).mp. [mp=title, abstract, heading word, table of contents, key concepts, original title, tests & measures] |
| 89. Congo/ |
| 90. (congo not ((democratic republic adj3 congo) or congo red or crimean-congo)).mp. [mp=title, abstract, heading word, table of contents, key concepts, original title, tests & measures] |
| 91. Cote d'Ivoire/ |
| 92. (Cote d'Ivoire or Ivory Coast).mp. [mp=title, abstract, heading word, table of contents, key concepts, original title, tests & measures] |
| 93. Ghana/ |
| 94. (Ghana or Gold Coast).mp. [mp=title, abstract, heading word, table of contents, key concepts, original title, tests & measures] |
| 95. Kenya/ |
| 96. kenya.mp. |
| 97. Lesotho/ |
| 98. (Lesotho or Basutoland).mp. [mp=title, abstract, heading word, table of contents, key concepts, original title, tests & measures] |
| 99. Mauritania/ |
| 100. Mauritania.mp. [mp=title, abstract, heading word, table of contents, key concepts, original title, tests & measures] |
| 101. Nigeria/ |
| 102. Nigeria.mp. [mp=title, abstract, heading word, table of contents, key concepts, original title, tests & measures] |
| 103. Atlantic Islands/ |
| 104. (sao tome adj2 principe).mp. [mp=title, abstract, heading word, table of contents, key concepts, original title, tests & measures] |
| 105. Sudan/ |
| 106. (Sudan not south sudan).mp. [mp=title, abstract, heading word, table of contents, key concepts, original title, tests & measures] |
| 107. Swaziland/ |
| 108. Swaziland.mp. [mp=title, abstract, heading word, table of contents, key concepts, original title, tests & measures] |
| 109. Zambia/ |
| 110. (Zambia or Northern Rhodesia).mp. [mp=title, abstract, heading word, table of contents, key concepts, original title, tests & measures] |
| 111. Angola/ |
| 112. Angola.mp. [mp=title, abstract, heading word, table of contents, key concepts, original title, tests & measures] |
| 113. Botswana/ |
| 114. (Botswana or Bechuanaland or Kalahari).mp. [mp=title, abstract, heading word, table of contents, key concepts, original title, tests & measures] |
| 115. Equatorial Guinea/ |
| 116. (Equatorial Guinea or Spanish Guinea).mp. |
| 117. Gabon/ |
| 118. (Gabon or Gabonese Republic).mp. [mp=title, abstract, heading word, table of contents, key concepts, original title, tests & measures] |
| 119. Mauritius/ |
| 120. (Mauritius or Agalega Islands).mp. [mp=title, abstract, heading word, table of contents, key concepts, original title, tests & measures] |
| 121. Namibia/ |
| 122. Namibia.mp. [mp=title, abstract, heading word, table of contents, key concepts, original title, tests & measures] |
| 123. South Africa/ |
| 124. South Africa.mp. [mp=title, abstract, heading word, table of contents, key concepts, original title, tests & measures] |
| 125. Seychelles/ |
| 126. Seychelles.mp. |
| 127. "africa south of the sahara"/ or africa, central/ or africa, eastern/ or africa, southern/ or africa, western/ |
| 128. ("africa south of the sahara" or sub-saharan africa or central africa or eastern africa or southern africa or western africa).mp. |
| 129. or/31-128 |
| 130. exp HIV/ or HIV.mp. or exp HIV Infections/ or exp HIV-1/ |
| 131. AIDS.mp. or Acquired Immunodeficiency Syndrome/ |
| 132. 130 or 131 |
| 133. 30 and 129 and 132 |
| 134. limit 133 to yr="2000 - 2019" |
