# Supplementary material for: Informing the measurement of wellbeing among young people living with HIV in sub-Saharan Africa for policy evaluations: a mixed-methods systematic review
Source: Health Qual Life Outcomes. 2020 May 5;18:120. doi: 10.1186/s12955-020-01352-w (PMC7201613; doi:10.1186/s12955-020-01352-w)
Supplement: Supplementary file 4 — Additional file 4. Search strategy- PsychInfo (OVID). [file 12955_2020_1352_MOESM4_ESM.docx]

Additional file 4- Search strategy (OVID PSYCH INFO)

| 1. Quality of Life.mp. or exp "Quality of Life"/ |
| --- |
| 2. health-related quality of life.mp. |
| 3. well being.mp. or exp Well Being/ |
| 4. wellbeing.mp. |
| 5. exp RELATIONSHIP SATISFACTION/ or exp SATISFACTION/ or exp MARITAL SATISFACTION/ or exp LIFE SATISFACTION/ or exp ROLE SATISFACTION/ or exp JOB SATISFACTION/ or exp NEED SATISFACTION/ |
| 6. exp NEGATIVE EMOTIONS/ or exp EMOTIONS/ or exp POSITIVE EMOTIONS/ |
| 7. exp PAIN/ |
| 8. exp ANXIETY/ |
| 9. exp ANGER/ |
| 10. exp LONELINESS/ |
| 11. mental health.mp. |
| 12. exp STRESS/ |
| 13. exp AUTONOMY/ |
| 14. exp "Resilience (Psychological)"/ |
| 15. exp COMPETENCE/ |
| 16. exp Emotional Control/ |
| 17. exp Self-Control/ |
| 18. exp Coping Behavior/ |
| 19. exp Interpersonal Relationships/ |
| 20. exp Self-Perception/ |
| 21. (subjective adj1 wellbeing).mp. [mp=title, abstract, heading word, table of contents, key concepts, original title, tests & measures] |
| 22. (subjective adj1 well-being).mp. [mp=title, abstract, heading word, table of contents, key concepts, original title, tests & measures] |
| 23. (psychological adj1 wellbeing).mp. [mp=title, abstract, heading word, table of contents, key concepts, original title, tests & measures] |
| 24. (psychological adj1 well-being).mp. [mp=title, abstract, heading word, table of contents, key concepts, original title, tests & measures] |
| 25. well-being.mp. |
| 26. exp FATIGUE/ |
| 27. exp Psychological Engagement/ |
| 28. exp Achievement/ |
| 29. exp Emotional Content/ |
| 30. exp Self-Esteem/ |
| 31. exp Positive Psychology/ |
| 32. (relational adj1 wellbeing).mp. [mp=title, abstract, heading word, table of contents, key concepts, original title, tests & measures] |
| 33. (relational adj1 well-being).mp. [mp=title, abstract, heading word, table of contents, key concepts, original title, tests & measures] |
| 34. happiness.mp. or exp HAPPINESS/ |
| 35. exp Emotional States/ |
| 36. exp GOALS/ |
| 37. exp NEEDS/ |
| 38. resources.mp. |
| 39. (lived adj1 experience$).mp. [mp=title, abstract, heading word, table of contents, key concepts, original title, tests & measures] |
| 40. or/1-39 |
| 41. (Benin or Dahomey).mp. [mp=title, abstract, heading word, table of contents, key concepts, original title, tests & measures] |
| 42. (Burkina Faso or Burkina Fasso or Upper Volta).mp. [mp=title, abstract, heading word, table of contents, key concepts, original title, tests & measures] |
| 43. Burundi.mp. [mp=title, abstract, heading word, table of contents, key concepts, original title, tests & measures] |
| 44. (Central African Republic or Ubangi-Shari).mp. [mp=title, abstract, heading word, table of contents, key concepts, original title, tests & measures] |
| 45. Chad.mp. [mp=title, abstract, heading word, table of contents, key concepts, original title, tests & measures] |
| 46. (Comoros or Comoro Islands or Mayotte or Iles Comores).mp. [mp=title, abstract, heading word, table of contents, key concepts, original title, tests & measures] |
| 47. ((democratic republic adj2 congo) or belgian congo or zaire).mp. [mp=title, abstract, heading word, table of contents, key concepts, original title, tests & measures] |
| 48. Eritrea.mp. [mp=title, abstract, heading word, table of contents, key concepts, original title, tests & measures] |
| 49. Ethiopia.mp. [mp=title, abstract, heading word, table of contents, key concepts, original title, tests & measures] |
| 50. Gambia.mp. [mp=title, abstract, heading word, table of contents, key concepts, original title, tests & measures] |
| 51. (Guinea not (New Guinea or Guinea Pig* or Guinea Fowl)).mp. [mp=title, abstract, heading word, table of contents, key concepts, original title, tests & measures] |
| 52. (Guinea-Bissau or Portuguese Guinea).mp. [mp=title, abstract, heading word, table of contents, key concepts, original title, tests & measures] |
| 53. Liberia.mp. [mp=title, abstract, heading word, table of contents, key concepts, original title, tests & measures] |
| 54. (Madagascar or Malagasy Republic).mp. [mp=title, abstract, heading word, table of contents, key concepts, original title, tests & measures] |
| 55. (Malawi or Nyasaland).mp. [mp=title, abstract, heading word, table of contents, key concepts, original title, tests & measures] |
| 56. Mali.mp. [mp=title, abstract, heading word, table of contents, key concepts, original title, tests & measures] |
| 57. (Mozambique or Portuguese East Africa).mp. [mp=title, abstract, heading word, table of contents, key concepts, original title, tests & measures] |
| 58. (Niger not (Aspergillus or Peptococcus or Schizothorax or Cruciferae or Gobius or Lasius or Agelastes or Melanosuchus or radish or Parastromateus or Orius or Apergillus or Parastromateus or Stomoxys)).mp. [mp=title, abstract, heading word, table of contents, key concepts, original title, tests & measures] |
| 59. (Rwanda or Ruanda).mp. [mp=title, abstract, heading word, table of contents, key concepts, original title, tests & measures] |
| 60. senegal.mp. |
| 61. Sierra Leone.mp. |
| 62. Somalia.mp. [mp=title, abstract, heading word, table of contents, key concepts, original title, tests & measures] |
| 63. south sudan.mp. |
| 64. (Tanzania or Zanzibar).mp. [mp=title, abstract, heading word, table of contents, key concepts, original title, tests & measures] |
| 65. (Togo or Togolese Republic).mp. [mp=title, abstract, heading word, table of contents, key concepts, original title, tests & measures] |
| 66. Uganda.mp. [mp=title, abstract, heading word, table of contents, key concepts, original title, tests & measures] |
| 67. (Zimbabwe or Rhodesia).mp. [mp=title, abstract, heading word, table of contents, key concepts, original title, tests & measures] |
| 68. Cameroon.mp. [mp=title, abstract, heading word, table of contents, key concepts, original title, tests & measures] |
| 69. (Cape Verde or Cabo Verde).mp. [mp=title, abstract, heading word, table of contents, key concepts, original title, tests & measures] |
| 70. (congo not ((democratic republic adj3 congo) or congo red or crimean-congo)).mp. [mp=title, abstract, heading word, table of contents, key concepts, original title, tests & measures] |
| 71. (Cote d'Ivoire or Ivory Coast).mp. [mp=title, abstract, heading word, table of contents, key concepts, original title, tests & measures] |
| 72. (Ghana or Gold Coast).mp. [mp=title, abstract, heading word, table of contents, key concepts, original title, tests & measures] |
| 73. kenya.mp. |
| 74. (Lesotho or Basutoland).mp. [mp=title, abstract, heading word, table of contents, key concepts, original title, tests & measures] |
| 75. Mauritania.mp. [mp=title, abstract, heading word, table of contents, key concepts, original title, tests & measures] |
| 76. Nigeria.mp. [mp=title, abstract, heading word, table of contents, key concepts, original title, tests & measures] |
| 77. (sao tome adj2 principe).mp. [mp=title, abstract, heading word, table of contents, key concepts, original title, tests & measures] |
| 78. (Sudan not south sudan).mp. [mp=title, abstract, heading word, table of contents, key concepts, original title, tests & measures] |
| 79. Swaziland.mp. [mp=title, abstract, heading word, table of contents, key concepts, original title, tests & measures] |
| 80. (Zambia or Northern Rhodesia).mp. [mp=title, abstract, heading word, table of contents, key concepts, original title, tests & measures] |
| 81. Angola.mp. [mp=title, abstract, heading word, table of contents, key concepts, original title, tests & measures] |
| 82. (Botswana or Bechuanaland or Kalahari).mp. [mp=title, abstract, heading word, table of contents, key concepts, original title, tests & measures] |
| 83. (Equatorial Guinea or Spanish Guinea).mp. |
| 84. (Gabon or Gabonese Republic).mp. [mp=title, abstract, heading word, table of contents, key concepts, original title, tests & measures] |
| 85. (Mauritius or Agalega Islands).mp. [mp=title, abstract, heading word, table of contents, key concepts, original title, tests & measures] |
| 86. Namibia.mp. [mp=title, abstract, heading word, table of contents, key concepts, original title, tests & measures] |
| 87. South Africa.mp. [mp=title, abstract, heading word, table of contents, key concepts, original title, tests & measures] |
| 88. Seychelles.mp. |
| 89. ("africa south of the sahara" or sub-saharan africa or central africa or eastern africa or southern africa or western africa).mp. |
| 90. exp HIV/ or HIV.mp. |
| 91. exp AIDS/ or AIDS.mp. |
| 92. or/41-89 |
| 93. or/90-91 |
| 94. 40 and 92 and 93 |
| 95. limit 94 to yr="2000 - 2019" |
