# Supplementary material for: Informing the measurement of wellbeing among young people living with HIV in sub-Saharan Africa for policy evaluations: a mixed-methods systematic review
Source: Health Qual Life Outcomes. 2020 May 5;18:120. doi: 10.1186/s12955-020-01352-w (PMC7201613; doi:10.1186/s12955-020-01352-w)
Supplement: Supplementary file 5 — Additional file 5. Search strategy- Econlit (OVID). [file 12955_2020_1352_MOESM5_ESM.docx]

Additional file 5- Search strategy (OVID Econlit)

| 1. Quality of Life.mp. |
| --- |
| 2. health-related quality of life.mp. |
| 3. well being.mp. |
| 4. wellbeing.mp. |
| 5. well-being.mp. |
| 6. (subjective adj1 wellbeing).mp. |
| 7. (subjective adj1 well-being).mp. |
| 8. (psychological adj1 wellbeing).mp. |
| 9. (psychological adj1 well-being).mp. |
| 10. (life adj1 satisfaction).mp. |
| 11. (health adj1 satisfaction).mp. |
| 12. (personal adj1 satisfaction).mp. |
| 13. satisfaction.mp. |
| 14. mood.mp. [mp=heading words, abstract, title, country as subject] |
| 15. emotions.mp. [mp=heading words, abstract, title, country as subject] |
| 16. affect.mp. [mp=heading words, abstract, title, country as subject] |
| 17. (positive adj1 affect).mp. [mp=heading words, abstract, title, country as subject] |
| 18. happiness.mp. [mp=heading words, abstract, title, country as subject] |
| 19. pleasure.mp. [mp=heading words, abstract, title, country as subject] |
| 20. (negative adj1 affect).mp. [mp=heading words, abstract, title, country as subject] |
| 21. pain.mp. [mp=heading words, abstract, title, country as subject] |
| 22. depression.mp. [mp=heading words, abstract, title, country as subject] |
| 23. anxiety.mp. [mp=heading words, abstract, title, country as subject] |
| 24. (mental adj1 health).mp. [mp=heading words, abstract, title, country as subject] |
| 25. (mental adj1 stress).mp. [mp=heading words, abstract, title, country as subject] |
| 26. resilience.mp. [mp=heading words, abstract, title, country as subject] |
| 27. (interpersonal adj1 relationships).mp. [mp=heading words, abstract, title, country as subject] |
| 28. (purpose adj2 life).mp. [mp=heading words, abstract, title, country as subject] |
| 29. (self adj1 acceptance).mp. [mp=heading words, abstract, title, country as subject] |
| 30. (self adj1 control).mp. [mp=heading words, abstract, title, country as subject] |
| 31. (personal adj1 growth).mp. [mp=heading words, abstract, title, country as subject] |
| 32. (self adj1 determined).mp. [mp=heading words, abstract, title, country as subject] |
| 33. (hedonic or hedonism).mp. |
| 34. tired.mp. |
| 35. (eudaimonic or eudaemonic or eudaimonism).mp. |
| 36. (positive adj1 psychology).mp. [mp=heading words, abstract, title, country as subject] |
| 37. (psychological adj1 functioning).mp. [mp=heading words, abstract, title, country as subject] |
| 38. "I3$".cc. |
| 39. "I1$".cc. |
| 40. "J1$".cc. |
| 41. "A14".cc. |
| 42. "Z1$".cc. |
| 43. (relational adj1 well-being).ti,ab,hw,ct. |
| 44. (relational adj1 wellbeing).ti,ab,hw,ct. |
| 45. (lived adj1 experience$).ti,ab,hw,ct. |
| 46. 1 or 2 or 3 or 4 or 5 or 6 or 7 or 8 or 9 or 10 or 11 or 12 or 13 or 14 or 15 or 16 or 17 or 18 or 19 or 20 or 21 or 22 or 23 or 24 or 25 or 26 or 27 or 28 or 29 or 30 or 31 or 32 or 33 or 34 or 35 or 36 or 37 or 38 or 39 or 40 or 41 or 42 or 43 or 44 or 45 |
| 47. (Benin or Dahomey).mp. [mp=heading words, abstract, title, country as subject] |
| 48. (Burkina Faso or Burkina Fasso or Upper Volta).mp. [mp=heading words, abstract, title, country as subject] |
| 49. Burundi.mp. [mp=heading words, abstract, title, country as subject] |
| 50. (Central African Republic or Ubangi-Shari).mp. [mp=heading words, abstract, title, country as subject] |
| 51. Chad.mp. [mp=heading words, abstract, title, country as subject] |
| 52. (Comoros or Comoro Islands or Mayotte or Iles Comores).mp. [mp=heading words, abstract, title, country as subject] |
| 53. ((democratic republic adj2 congo) or belgian congo or zaire).mp. [mp=heading words, abstract, title, country as subject] |
| 54. Eritrea.mp. [mp=heading words, abstract, title, country as subject] |
| 55. Ethiopia.mp. [mp=heading words, abstract, title, country as subject] |
| 56. Gambia.mp. [mp=heading words, abstract, title, country as subject] |
| 57. (Guinea not (New Guinea or Guinea Pig* or Guinea Fowl)).mp. [mp=heading words, abstract, title, country as subject] |
| 58. (Guinea-Bissau or Portuguese Guinea).mp. [mp=heading words, abstract, title, country as subject] |
| 59. Liberia.mp. [mp=heading words, abstract, title, country as subject] |
| 60. (Madagascar or Malagasy Republic).mp. [mp=heading words, abstract, title, country as subject] |
| 61. (Malawi or Nyasaland).mp. [mp=heading words, abstract, title, country as subject] |
| 62. Mali.mp. [mp=heading words, abstract, title, country as subject] |
| 63. (Mozambique or Portuguese East Africa).mp. [mp=heading words, abstract, title, country as subject] |
| 64. (Niger not (Aspergillus or Peptococcus or Schizothorax or Cruciferae or Gobius or Lasius or Agelastes or Melanosuchus or radish or Parastromateus or Orius or Apergillus or Parastromateus or Stomoxys)).mp. [mp=heading words, abstract, title, country as subject] |
| 65. (Rwanda or Ruanda).mp. [mp=heading words, abstract, title, country as subject] |
| 66. senegal.mp. |
| 67. Sierra Leone.mp. |
| 68. Somalia.mp. [mp=heading words, abstract, title, country as subject] |
| 69. south sudan.mp. |
| 70. (Tanzania or Zanzibar).mp. [mp=heading words, abstract, title, country as subject] |
| 71. (Togo or Togolese Republic).mp. [mp=heading words, abstract, title, country as subject] |
| 72. Uganda.mp. [mp=heading words, abstract, title, country as subject] |
| 73. (Zimbabwe or Rhodesia).mp. [mp=heading words, abstract, title, country as subject] |
| 74. Cameroon.mp. [mp=heading words, abstract, title, country as subject] |
| 75. (Cape Verde or Cabo Verde).mp. [mp=heading words, abstract, title, country as subject] |
| 76. (congo not ((democratic republic adj3 congo) or congo red or crimean-congo)).mp. [mp=heading words, abstract, title, country as subject] |
| 77. (Cote d'Ivoire or Ivory Coast).mp. [mp=heading words, abstract, title, country as subject] |
| 78. (Ghana or Gold Coast).mp. [mp=heading words, abstract, title, country as subject] |
| 79. kenya.mp. |
| 80. (Lesotho or Basutoland).mp. [mp=heading words, abstract, title, country as subject] |
| 81. Mauritania.mp. [mp=heading words, abstract, title, country as subject] |
| 82. Nigeria.mp. [mp=heading words, abstract, title, country as subject] |
| 83. (sao tome adj2 principe).mp. [mp=heading words, abstract, title, country as subject] |
| 84. (Sudan not south sudan).mp. [mp=heading words, abstract, title, country as subject] |
| 85. Swaziland.mp. [mp=heading words, abstract, title, country as subject] |
| 86. (Zambia or Northern Rhodesia).mp. [mp=heading words, abstract, title, country as subject] |
| 87. Angola.mp. [mp=heading words, abstract, title, country as subject] |
| 88. (Botswana or Bechuanaland or Kalahari).mp. [mp=heading words, abstract, title, country as subject] |
| 89. (Equatorial Guinea or Spanish Guinea).mp. |
| 90. (Gabon or Gabonese Republic).mp. [mp=heading words, abstract, title, country as subject] |
| 91. (Mauritius or Agalega Islands).mp. [mp=heading words, abstract, title, country as subject] |
| 92. Namibia.mp. [mp=heading words, abstract, title, country as subject] |
| 93. South Africa.mp. [mp=heading words, abstract, title, country as subject] |
| 94. Seychelles.mp. |
| 95. ("africa south of the sahara" or sub-saharan africa or central africa or eastern africa or southern africa or western africa).mp. |
| 96. exp HIV/ or HIV.mp. |
| 97. exp AIDS/ or AIDS.mp. |
| 98. or/47-95 |
| 99. or/96-97 |
| 100. 46 and 98 and 99 |
| 101. limit 100 to yr="2000-2019" |
