# Supplementary material for: Informing the measurement of wellbeing among young people living with HIV in sub-Saharan Africa for policy evaluations: a mixed-methods systematic review
Source: Health Qual Life Outcomes. 2020 May 5;18:120. doi: 10.1186/s12955-020-01352-w (PMC7201613; doi:10.1186/s12955-020-01352-w)
Supplement: Supplementary file 6 — Additional file 6. Search strategy- AfricaWide (EMBASE). [file 12955_2020_1352_MOESM6_ESM.docx]

Additional file 6: Search strategy (EBSCO Africa Wide)

| # Query Limiters/Expanders Last Run Via Results Action |
| --- |
| S85 S78 AND S83 AND S84 Limiters - Scholarly (Peer Reviewed) Journals; Publication Type: Journal Article; Document Type: Article; Year Published: 2000-2019 |
| Search modes - Boolean/Phrase Interface - EBSCOhost Research Databases |
| Search Screen - Advanced Search |
| Database - Africa-Wide Information 702 Edit S85 |
| S84 S81 OR S82 Search modes - Boolean/Phrase Interface - EBSCOhost Research Databases |
| Search Screen - Advanced Search |
| Database - Africa-Wide Information 153,879 Edit S84 |
| S83 S79 OR S80 Search modes - Boolean/Phrase Interface - EBSCOhost Research Databases |
| Search Screen - Advanced Search |
| Database - Africa-Wide Information 1,996,682 Edit S83 |
| S82 AIDS or Acquired Immunodeficiency Syndrome Search modes - Boolean/Phrase Interface - EBSCOhost Research Databases |
| Search Screen - Advanced Search |
| Database - Africa-Wide Information 106,965 Edit S82 |
| S81 HIV or HIV-positive or HIV-Positive or HIV-infected or HIV/AIDS or PLHIV Search modes - Boolean/Phrase Interface - EBSCOhost Research Databases |
| Search Screen - Advanced Search |
| Database - Africa-Wide Information 126,100 Edit S81 |
| S80 sub-saharan africa or sub saharan africa or subsaharan africa Search modes - Boolean/Phrase Interface - EBSCOhost Research Databases |
| Search Screen - Advanced Search |
| Database - Africa-Wide Information 49,785 Edit S80 |
| S79 AB Angola or Benin or Botswana or Burkina Faso or Burundi or Cameroon or Cape Verde or Central African Republic or CHAD or Comoros or Congo or Congo Democratic Republic or Djibouti or Equatorial Guinea or Eritrea or Ethiopia or Gabon or Gambia or Ghana or Guinea or Guinea-Bissau or Cote d'Ivoire or Ivory Coast or Kenya or Lesotho or Liberia or Madagascar or Malawi or Mali or Mozambique or Namibia or Niger or Nigeria or Sao tome and Principe or Rwanda or Senegal or Seychelles or Sierra Leone or Somalia or South Africa or South Sudan or Sudan or Swaziland or Tanzania or Togo or Uganda or Zambia or Zimbabwe Search modes - Boolean/Phrase Interface - EBSCOhost Research Databases |
| Search Screen - Advanced Search |
| Database - Africa-Wide Information 1,977,965 Edit S79 |
| S78 S40 OR S41 OR S42 OR S43 OR S44 OR S45 OR S46 OR S47 OR S48 OR S49 OR S50 OR S51 OR S52 OR S53 OR S54 OR S55 OR S56 OR S57 OR S58 OR S59 OR S60 OR S61 OR S62 OR S63 OR S64 OR S65 OR S66 OR S67 OR S68 OR S69 OR S70 OR S71 OR S72 OR S73 OR S74 OR S75 OR S76 OR S77 Search modes - Boolean/Phrase Interface - EBSCOhost Research Databases |
| Search Screen - Advanced Search |
| Database - Africa-Wide Information Display Edit S78 |
| S77 lived AND experience$ Limiters - Scholarly (Peer Reviewed) Journals; Publication Type: Journal Article; Document Type: Article |
| Search modes - Boolean/Phrase Interface - EBSCOhost Research Databases |
| Search Screen - Advanced Search |
| Database - Africa-Wide Information Display Edit S77 |
| S76 relational AND wellbeing Limiters - Scholarly (Peer Reviewed) Journals; Publication Type: Journal Article; Document Type: Article |
| Search modes - Boolean/Phrase Interface - EBSCOhost Research Databases |
| Search Screen - Advanced Search |
| Database - Africa-Wide Information Display Edit S76 |
| S75 relational AND wellbeing Limiters - Scholarly (Peer Reviewed) Journals; Publication Type: Journal Article; Document Type: Article |
| Search modes - Boolean/Phrase Interface - EBSCOhost Research Databases |
| Search Screen - Advanced Search |
| Database - Africa-Wide Information Display Edit S75 |
| S74 AB accomplishments Search modes - Boolean/Phrase Interface - EBSCOhost Research Databases |
| Search Screen - Advanced Search |
| Database - Africa-Wide Information Display Edit S74 |
| S73 AB psychological AND functioning Search modes - Boolean/Phrase Interface - EBSCOhost Research Databases |
| Search Screen - Advanced Search |
| Database - Africa-Wide Information Display Edit S73 |
| S72 AB psychological AND engagement Search modes - Boolean/Phrase Interface - EBSCOhost Research Databases |
| Search Screen - Advanced Search |
| Database - Africa-Wide Information Display Edit S72 |
| S71 AB depressed or depression Search modes - Boolean/Phrase Interface - EBSCOhost Research Databases |
| Search Screen - Advanced Search |
| Database - Africa-Wide Information Display Edit S71 |
| S70 AB tired or fatigue Search modes - Boolean/Phrase Interface - EBSCOhost Research Databases |
| Search Screen - Advanced Search |
| Database - Africa-Wide Information Display Edit S70 |
| S69 AB adaptation, psychological Search modes - Boolean/Phrase Interface - EBSCOhost Research Databases |
| Search Screen - Advanced Search |
| Database - Africa-Wide Information Display Edit S69 |
| S68 AB resilience, psychological Search modes - Boolean/Phrase Interface - EBSCOhost Research Databases |
| Search Screen - Advanced Search |
| Database - Africa-Wide Information Display Edit S68 |
| S67 AB mental AND competency Search modes - Boolean/Phrase Interface - EBSCOhost Research Databases |
| Search Screen - Advanced Search |
| Database - Africa-Wide Information Display Edit S67 |
| S66 AB autonomy Limiters - Scholarly (Peer Reviewed) Journals |
| Search modes - Boolean/Phrase Interface - EBSCOhost Research Databases |
| Search Screen - Advanced Search |
| Database - Africa-Wide Information Display Edit S66 |
| S65 AB personal AND growth Limiters - Scholarly (Peer Reviewed) Journals |
| Search modes - Boolean/Phrase Interface - EBSCOhost Research Databases |
| Search Screen - Advanced Search |
| Database - Africa-Wide Information Display Edit S65 |
| S64 AB self AND control Limiters - Scholarly (Peer Reviewed) Journals |
| Search modes - Boolean/Phrase Interface - EBSCOhost Research Databases |
| Search Screen - Advanced Search |
| Database - Africa-Wide Information Display Edit S64 |
| S63 AB self AND acceptance Limiters - Scholarly (Peer Reviewed) Journals |
| Search modes - Boolean/Phrase Interface - EBSCOhost Research Databases |
| Search Screen - Advanced Search |
| Database - Africa-Wide Information Display Edit S63 |
| S62 AB purpose in life Limiters - Scholarly (Peer Reviewed) Journals |
| Search modes - Boolean/Phrase Interface - EBSCOhost Research Databases |
| Search Screen - Advanced Search |
| Database - Africa-Wide Information Display Edit S62 |
| S61 AB positive AND relationships Limiters - Scholarly (Peer Reviewed) Journals |
| Search modes - Boolean/Phrase Interface - EBSCOhost Research Databases |
| Search Screen - Advanced Search |
| Database - Africa-Wide Information Display Edit S61 |
| S60 AB interpersonal AND relationships Limiters - Scholarly (Peer Reviewed) Journals |
| Search modes - Boolean/Phrase Interface - EBSCOhost Research Databases |
| Search Screen - Advanced Search |
| Database - Africa-Wide Information Display Edit S60 |
| S59 AB environmental mastery Limiters - Scholarly (Peer Reviewed) Journals |
| Search modes - Boolean/Phrase Interface - EBSCOhost Research Databases |
| Search Screen - Advanced Search |
| Database - Africa-Wide Information Display Edit S59 |
| S58 mental AND health Limiters - Scholarly (Peer Reviewed) Journals |
| Search modes - Boolean/Phrase Interface - EBSCOhost Research Databases |
| Search Screen - Advanced Search |
| Database - Africa-Wide Information Display Edit S58 |
| S57 AB anxiety or worry Limiters - Scholarly (Peer Reviewed) Journals |
| Search modes - Boolean/Phrase Interface - EBSCOhost Research Databases |
| Search Screen - Advanced Search |
| Database - Africa-Wide Information Display Edit S57 |
| S56 AB pain Limiters - Scholarly (Peer Reviewed) Journals |
| Search modes - Boolean/Phrase Interface - EBSCOhost Research Databases |
| Search Screen - Advanced Search |
| Database - Africa-Wide Information Display Edit S56 |
| S55 AB happiness Limiters - Scholarly (Peer Reviewed) Journals |
| Search modes - Boolean/Phrase Interface - EBSCOhost Research Databases |
| Search Screen - Advanced Search |
| Database - Africa-Wide Information Display Edit S55 |
| S54 AB negative AND emotion Limiters - Scholarly (Peer Reviewed) Journals |
| Search modes - Boolean/Phrase Interface - EBSCOhost Research Databases |
| Search Screen - Advanced Search |
| Database - Africa-Wide Information Display Edit S54 |
| S53 AB negative AND affect Limiters - Scholarly (Peer Reviewed) Journals |
| Search modes - Boolean/Phrase Interface - EBSCOhost Research Databases |
| Search Screen - Advanced Search |
| Database - Africa-Wide Information Display Edit S53 |
| S52 AB positive AND emotion Limiters - Scholarly (Peer Reviewed) Journals |
| Search modes - Boolean/Phrase Interface - EBSCOhost Research Databases |
| Search Screen - Advanced Search |
| Database - Africa-Wide Information Display Edit S52 |
| S51 AB positive AND affect Limiters - Scholarly (Peer Reviewed) Journals |
| Search modes - Boolean/Phrase Interface - EBSCOhost Research Databases |
| Search Screen - Advanced Search |
| Database - Africa-Wide Information Display Edit S51 |
| S50 life AND satisfaction Limiters - Scholarly (Peer Reviewed) Journals |
| Search modes - Boolean/Phrase Interface - EBSCOhost Research Databases |
| Search Screen - Advanced Search |
| Database - Africa-Wide Information Display Edit S50 |
| S49 psychological AND well-being Limiters - Scholarly (Peer Reviewed) Journals |
| Search modes - Boolean/Phrase Interface - EBSCOhost Research Databases |
| Search Screen - Advanced Search |
| Database - Africa-Wide Information Display Edit S49 |
| S48 psychological AND well-being Limiters - Scholarly (Peer Reviewed) Journals |
| Search modes - Boolean/Phrase Interface - EBSCOhost Research Databases |
| Search Screen - Advanced Search |
| Database - Africa-Wide Information Display Edit S48 |
| S47 psychological AND wellbeing Limiters - Scholarly (Peer Reviewed) Journals |
| Search modes - Boolean/Phrase Interface - EBSCOhost Research Databases |
| Search Screen - Advanced Search |
| Database - Africa-Wide Information Display Edit S47 |
| S46 subjective AND well-being Limiters - Scholarly (Peer Reviewed) Journals |
| Search modes - Boolean/Phrase Interface - EBSCOhost Research Databases |
| Search Screen - Advanced Search |
| Database - Africa-Wide Information Display Edit S46 |
| S45 subjective AND wellbeing Limiters - Scholarly (Peer Reviewed) Journals |
| Search modes - Boolean/Phrase Interface - EBSCOhost Research Databases |
| Search Screen - Advanced Search |
| Database - Africa-Wide Information Display Edit S45 |
| S44 wellbeing or well-being or well being Limiters - Scholarly (Peer Reviewed) Journals |
| Search modes - Boolean/Phrase Interface - EBSCOhost Research Databases |
| Search Screen - Advanced Search |
| Database - Africa-Wide Information Display Edit S44 |
| S43 hrqol or HRQOL or HRQoL or HRQol or HRqol Limiters - Scholarly (Peer Reviewed) Journals |
| Search modes - Boolean/Phrase Interface - EBSCOhost Research Databases |
| Search Screen - Advanced Search |
| Database - Africa-Wide Information Display Edit S43 |
| S42 health-related quality of life or health related quality of life Limiters - Scholarly (Peer Reviewed) Journals |
| Search modes - Boolean/Phrase Interface - EBSCOhost Research Databases |
| Search Screen - Advanced Search |
| Database - Africa-Wide Information Display Edit S42 |
| S41 QOL or Qol or QoL or qol Limiters - Scholarly (Peer Reviewed) Journals |
| Search modes - Boolean/Phrase Interface - EBSCOhost Research Databases |
| Search Screen - Advanced Search |
| Database - Africa-Wide Information Display Edit S41 |
| S40 quality of life Limiters - Scholarly (Peer Reviewed) Journals |
| Search modes - Boolean/Phrase Interface - EBSCOhost Research Databases |
| Search Screen - Advanced Search |
| Database - Africa-Wide Information Display Edit S40 |
| S39 S1 OR S2 OR S3 OR S4 OR S5 OR S6 OR S7 OR S8 OR S9 OR S10 OR S11 OR S12 OR S13 OR S14 OR S15 OR S16 OR S17 OR S18 OR S19 OR S20 OR S21 OR S22 OR S23 OR S24 OR S25 OR S26 OR S27 OR S28 OR S29 OR S30 OR S31 OR S32 OR S33 OR S34 OR S35 OR S36 OR S37 OR S38 Search modes - Boolean/Phrase Interface - EBSCOhost Research Databases |
| Search Screen - Advanced Search |
| Database - Africa-Wide Information 56,621 Edit S39 |
| S38 lived AND experience$ Limiters - Scholarly (Peer Reviewed) Journals; Publication Type: Journal Article; Document Type: Article |
| Search modes - Boolean/Phrase Interface - EBSCOhost Research Databases |
| Search Screen - Advanced Search |
| Database - Africa-Wide Information 132 Edit S38 |
| S37 relational AND wellbeing Limiters - Scholarly (Peer Reviewed) Journals; Publication Type: Journal Article; Document Type: Article |
| Search modes - Boolean/Phrase Interface - EBSCOhost Research Databases |
| Search Screen - Advanced Search |
| Database - Africa-Wide Information 2 Edit S37 |
| S36 relational AND wellbeing Limiters - Scholarly (Peer Reviewed) Journals; Publication Type: Journal Article; Document Type: Article |
| Search modes - Boolean/Phrase Interface - EBSCOhost Research Databases |
| Search Screen - Advanced Search |
| Database - Africa-Wide Information 2 Edit S36 |
| S35 AB accomplishments Search modes - Boolean/Phrase Interface - EBSCOhost Research Databases |
| Search Screen - Advanced Search |
| Database - Africa-Wide Information 414 Edit S35 |
| S34 AB psychological AND functioning Search modes - Boolean/Phrase Interface - EBSCOhost Research Databases |
| Search Screen - Advanced Search |
| Database - Africa-Wide Information 650 Edit S34 |
| S33 AB psychological AND engagement Search modes - Boolean/Phrase Interface - EBSCOhost Research Databases |
| Search Screen - Advanced Search |
| Database - Africa-Wide Information 122 Edit S33 |
| S32 AB depressed or depression Search modes - Boolean/Phrase Interface - EBSCOhost Research Databases |
| Search Screen - Advanced Search |
| Database - Africa-Wide Information 16,734 Edit S32 |
| S31 AB tired or fatigue Search modes - Boolean/Phrase Interface - EBSCOhost Research Databases |
| Search Screen - Advanced Search |
| Database - Africa-Wide Information 3,938 Edit S31 |
| S30 AB adaptation, psychological Search modes - Boolean/Phrase Interface - EBSCOhost Research Databases |
| Search Screen - Advanced Search |
| Database - Africa-Wide Information 36 Edit S30 |
| S29 AB resilience, psychological Search modes - Boolean/Phrase Interface - EBSCOhost Research Databases |
| Search Screen - Advanced Search |
| Database - Africa-Wide Information 38 Edit S29 |
| S28 AB mental AND competency Search modes - Boolean/Phrase Interface - EBSCOhost Research Databases |
| Search Screen - Advanced Search |
| Database - Africa-Wide Information 89 Edit S28 |
| S27 AB autonomy Limiters - Scholarly (Peer Reviewed) Journals |
| Search modes - Boolean/Phrase Interface - EBSCOhost Research Databases |
| Search Screen - Advanced Search |
| Database - Africa-Wide Information 1,098 Edit S27 |
| S26 AB personal AND growth Limiters - Scholarly (Peer Reviewed) Journals |
| Search modes - Boolean/Phrase Interface - EBSCOhost Research Databases |
| Search Screen - Advanced Search |
| Database - Africa-Wide Information 289 Edit S26 |
| S25 AB self AND control Limiters - Scholarly (Peer Reviewed) Journals |
| Search modes - Boolean/Phrase Interface - EBSCOhost Research Databases |
| Search Screen - Advanced Search |
| Database - Africa-Wide Information 2,849 Edit S25 |
| S24 AB self AND acceptance Limiters - Scholarly (Peer Reviewed) Journals |
| Search modes - Boolean/Phrase Interface - EBSCOhost Research Databases |
| Search Screen - Advanced Search |
| Database - Africa-Wide Information 366 Edit S24 |
| S23 AB purpose in life Limiters - Scholarly (Peer Reviewed) Journals |
| Search modes - Boolean/Phrase Interface - EBSCOhost Research Databases |
| Search Screen - Advanced Search |
| Database - Africa-Wide Information 198 Edit S23 |
| S22 AB positive AND relationships Limiters - Scholarly (Peer Reviewed) Journals |
| Search modes - Boolean/Phrase Interface - EBSCOhost Research Databases |
| Search Screen - Advanced Search |
| Database - Africa-Wide Information 4,722 Edit S22 |
| S21 AB interpersonal AND relationships Limiters - Scholarly (Peer Reviewed) Journals |
| Search modes - Boolean/Phrase Interface - EBSCOhost Research Databases |
| Search Screen - Advanced Search |
| Database - Africa-Wide Information 367 Edit S21 |
| S20 AB environmental mastery Limiters - Scholarly (Peer Reviewed) Journals |
| Search modes - Boolean/Phrase Interface - EBSCOhost Research Databases |
| Search Screen - Advanced Search |
| Database - Africa-Wide Information 8 Edit S20 |
| S19 mental AND health Limiters - Scholarly (Peer Reviewed) Journals |
| Search modes - Boolean/Phrase Interface - EBSCOhost Research Databases |
| Search Screen - Advanced Search |
| Database - Africa-Wide Information 9,474 Edit S19 |
| S18 AB anxiety or worry Limiters - Scholarly (Peer Reviewed) Journals |
| Search modes - Boolean/Phrase Interface - EBSCOhost Research Databases |
| Search Screen - Advanced Search |
| Database - Africa-Wide Information 3,439 Edit S18 |
| S17 AB pain Limiters - Scholarly (Peer Reviewed) Journals |
| Search modes - Boolean/Phrase Interface - EBSCOhost Research Databases |
| Search Screen - Advanced Search |
| Database - Africa-Wide Information 9,353 Edit S17 |
| S16 AB happiness Limiters - Scholarly (Peer Reviewed) Journals |
| Search modes - Boolean/Phrase Interface - EBSCOhost Research Databases |
| Search Screen - Advanced Search |
| Database - Africa-Wide Information 187 Edit S16 |
| S15 AB negative AND emotion Limiters - Scholarly (Peer Reviewed) Journals |
| Search modes - Boolean/Phrase Interface - EBSCOhost Research Databases |
| Search Screen - Advanced Search |
| Database - Africa-Wide Information 120 Edit S15 |
| S14 AB negative AND affect Limiters - Scholarly (Peer Reviewed) Journals |
| Search modes - Boolean/Phrase Interface - EBSCOhost Research Databases |
| Search Screen - Advanced Search |
| Database - Africa-Wide Information 1,299 Edit S14 |
| S13 AB positive AND emotion Limiters - Scholarly (Peer Reviewed) Journals |
| Search modes - Boolean/Phrase Interface - EBSCOhost Research Databases |
| Search Screen - Advanced Search |
| Database - Africa-Wide Information 165 Edit S13 |
| S12 AB positive AND affect Limiters - Scholarly (Peer Reviewed) Journals |
| Search modes - Boolean/Phrase Interface - EBSCOhost Research Databases |
| Search Screen - Advanced Search |
| Database - Africa-Wide Information 1,642 Edit S12 |
| S11 life AND satisfaction Limiters - Scholarly (Peer Reviewed) Journals |
| Search modes - Boolean/Phrase Interface - EBSCOhost Research Databases |
| Search Screen - Advanced Search |
| Database - Africa-Wide Information 571 Edit S11 |
| S10 psychological AND well-being Limiters - Scholarly (Peer Reviewed) Journals |
| Search modes - Boolean/Phrase Interface - EBSCOhost Research Databases |
| Search Screen - Advanced Search |
| Database - Africa-Wide Information 568 Edit S10 |
| S9 psychological AND well-being Limiters - Scholarly (Peer Reviewed) Journals |
| Search modes - Boolean/Phrase Interface - EBSCOhost Research Databases |
| Search Screen - Advanced Search |
| Database - Africa-Wide Information 568 Edit S9 |
| S8 psychological AND wellbeing Limiters - Scholarly (Peer Reviewed) Journals |
| Search modes - Boolean/Phrase Interface - EBSCOhost Research Databases |
| Search Screen - Advanced Search |
| Database - Africa-Wide Information 228 Edit S8 |
| S7 subjective AND well-being Limiters - Scholarly (Peer Reviewed) Journals |
| Search modes - Boolean/Phrase Interface - EBSCOhost Research Databases |
| Search Screen - Advanced Search |
| Database - Africa-Wide Information 113 Edit S7 |
| S6 subjective AND wellbeing Limiters - Scholarly (Peer Reviewed) Journals |
| Search modes - Boolean/Phrase Interface - EBSCOhost Research Databases |
| Search Screen - Advanced Search |
| Database - Africa-Wide Information 37 Edit S6 |
| S5 wellbeing or well-being or well being Limiters - Scholarly (Peer Reviewed) Journals |
| Search modes - Boolean/Phrase Interface - EBSCOhost Research Databases |
| Search Screen - Advanced Search |
| Database - Africa-Wide Information 4,010 Edit S5 |
| S4 hrqol or HRQOL or HRQoL or HRQol or HRqol Limiters - Scholarly (Peer Reviewed) Journals |
| Search modes - Boolean/Phrase Interface - EBSCOhost Research Databases |
| Search Screen - Advanced Search |
| Database - Africa-Wide Information 225 Edit S4 |
| S3 health-related quality of life or health related quality of life Limiters - Scholarly (Peer Reviewed) Journals |
| Search modes - Boolean/Phrase Interface - EBSCOhost Research Databases |
| Search Screen - Advanced Search |
| Database - Africa-Wide Information 573 Edit S3 |
| S2 QOL or Qol or QoL or qol Limiters - Scholarly (Peer Reviewed) Journals |
| Search modes - Boolean/Phrase Interface - EBSCOhost Research Databases |
| Search Screen - Advanced Search |
| Database - Africa-Wide Information 497 Edit S2 |
| S1 quality of life Limiters - Scholarly (Peer Reviewed) Journals |
| Search modes - Boolean/Phrase Interface - EBSCOhost Research Databases |
| Search Screen - Advanced Search |
| Database - Africa-Wide Information 4,978 |
