# Supplementary material for: Informing the measurement of wellbeing among young people living with HIV in sub-Saharan Africa for policy evaluations: a mixed-methods systematic review
Source: Health Qual Life Outcomes. 2020 May 5;18:120. doi: 10.1186/s12955-020-01352-w (PMC7201613; doi:10.1186/s12955-020-01352-w)
Supplement: Supplementary file 7 — Additional file 7. Search strategy- Web of Science. [file 12955_2020_1352_MOESM7_ESM.docx]

Additional file 7: Search strategy (Web of Science)

| Set |
| --- |
| Run Search Web of Science Core Collection |
| Search History - " Wellbeing SR_2019" |
| #36 #35 AND #32 |
| DocType=All document types; Language=All languages; |
| #35 #34 AND #33 AND #27 |
| DocType=All document types; Language=All languages; |
| #34 #31 OR #30 |
| DocType=All document types; Language=All languages; |
| #33 #29 OR #28 |
| DocType=All document types; Language=All languages; |
| #32 (((SU=(Arts Humanities) OR SU=(Life Sciences Biomedicine) OR SU=(Social Sciences)))) AND DOCUMENT TYPES: (Article) |
| DocType=All document types; Language=All languages; |
| #31 (((TS=(AIDS) OR TS=(Acquired Immunodeficiency Syndrome)))) AND DOCUMENT TYPES: (Article) |
| DocType=All document types; Language=All languages; |
| #30 (((TS=(HIV) OR TS=(HIV-positive) OR TS=(HIV-infect*) OR TS=(HIV/AIDS) OR TS=(PLHIV)))) AND DOCUMENT TYPES: (Article) |
| DocType=All document types; Language=All languages; |
| #29 ((TS=(sub-Saharan AND Africa))) AND DOCUMENT TYPES: (Article) |
| DocType=All document types; Language=All languages; |
| #28 ((CU=(Angola) OR CU=(Benin) OR CU=(Botswana) OR CU=(Burkina Faso) OR CU=(Burundi) OR CU=(Cameroon) OR CU=(Cape Verde) OR CU=(Central African Republic) OR CU=(CHAD) OR CU=(Comoros) OR CU=(Congo) OR CU=(Congo Democratic Republic) OR CU=(Djibouti) OR CU=(Equatorial Guinea) OR CU=(Eritrea) OR CU=(Ethiopia) OR CU=(Gabon) OR CU=(Gambia) OR CU=(Ghana) OR CU=(Guinea) OR CU=(Guinea-Bissau) OR CU=(Cote d'Ivoire) OR CU=(Ivory Coast) OR CU=(Kenya) OR CU=(Lesotho) OR CU=(Liberia) OR CU=(Madagascar) OR CU=(Malawi) OR CU=(Mali) OR CU=(Mozambique) OR CU=(Namibia) OR CU=(Niger) OR CU=(Nigeria) OR CU=(Sao tome and Principe) OR CU=(Rwanda) OR CU=(Senegal) OR CU=(Seychelles) OR CU=(Sierra Leone) OR CU=(Somalia) OR CU=(South Africa) OR CU=(South Sudan) OR CU=(Sudan) OR CU=(Swaziland) OR CU=(Tanzania) OR CU=(Togo) OR CU=(Uganda) OR CU=(Zambia) OR CU=(Zimbabwe))) AND DOCUMENT TYPES: (Article) |
| DocType=All document types; Language=All languages; |
| #27 #26 OR #25 OR #24 OR #23 OR #22 OR #21 OR #20 OR #19 OR #18 OR #17 OR #16 OR #15 OR #14 OR #13 OR #12 OR #11 OR #10 OR #9 OR #8 OR #7 OR #6 OR #5 OR #4 OR #3 OR #2 OR #1 |
| DocType=All document types; Language=All languages; |
| #26 (TI=(lived and experience$)) AND DOCUMENT TYPES: (Article) |
| DocType=All document types; Language=All languages; |
| #25 (TS=(relational and wellbeing) OR TS=(relational and well-being)) AND DOCUMENT TYPES: (Article) |
| DocType=All document types; Language=All languages; |
| #24 (TS=(psychological AND functioning)) AND DOCUMENT TYPES: (Article) |
| DocType=All document types; Language=All languages; |
| #23 (TS=(Psychological AND engagement)) AND DOCUMENT TYPES: (Article) |
| DocType=All document types; Language=All languages; |
| #22 (TS=(tired or fatigue)) AND DOCUMENT TYPES: (Article) |
| DocType=All document types; Language=All languages; |
| #21 (TS=(personal AND growth)) AND DOCUMENT TYPES: (Article) |
| DocType=All document types; Language=All languages; |
| #20 (TS=(self AND control)) AND DOCUMENT TYPES: (Article) |
| DocType=All document types; Language=All languages; |
| #19 (TS=(self AND acceptance)) AND DOCUMENT TYPES: (Article) |
| DocType=All document types; Language=All languages; |
| #18 (TS=(purpose in life)) AND DOCUMENT TYPES: (Article) |
| DocType=All document types; Language=All languages; |
| #17 (TS=(interpersonal AND relationships)) AND DOCUMENT TYPES: (Article) |
| DocType=All document types; Language=All languages; |
| #16 (TS=(resilience)) AND DOCUMENT TYPES: (Article) |
| DocType=All document types; Language=All languages; |
| #15 (TS=(mental AND health)) AND DOCUMENT TYPES: (Article) |
| DocType=All document types; Language=All languages; |
| #14 (TS=(anxiety)) AND DOCUMENT TYPES: (Article) |
| DocType=All document types; Language=All languages; |
| #13 (TS=(depression)) AND DOCUMENT TYPES: (Article) |
| DocType=All document types; Language=All languages; |
| #12 (TS=(pain)) AND DOCUMENT TYPES: (Article) |
| DocType=All document types; Language=All languages; |
| #11 (TS=(negative AND affect) OR TS=(negative AND emotion*) OR TS=(unpleasant AND affect)) AND DOCUMENT TYPES: (Article) |
| DocType=All document types; Language=All languages; |
| #10 (TS=(happiness) OR TS=(happy) OR TS=(pleasure)) AND DOCUMENT TYPES: (Article) |
| DocType=All document types; Language=All languages; |
| #9 (TS=(positive AND affect) OR TS=(positive AND emotion*) OR TS=(pleasant AND affect)) AND DOCUMENT TYPES: (Article) |
| DocType=All document types; Language=All languages; |
| #8 (TS=(mood) OR TS=(emotion*) OR TS=(emotion AND state*)) AND DOCUMENT TYPES: (Article) |
| DocType=All document types; Language=All languages; |
| #7 (TS=(life AND satisfaction) OR TS=(health AND satisfaction) OR TS=(personal AND satisfaction) OR TS=(satisfaction)) AND DOCUMENT TYPES: (Article) |
| DocType=All document types; Language=All languages; |
| #6 (TS=(psychological AND wellbeing) OR TS=(psychological AND well-being)) AND DOCUMENT TYPES: (Article) |
| DocType=All document types; Language=All languages; |
| #5 (TS=(subjective AND wellbeing) OR TS=(subjective AND well-being)) AND DOCUMENT TYPES: (Article) |
| DocType=All document types; Language=All languages; |
| #4 ((TS=(wellbeing) OR TS=(well-being) OR TS=(well being))) AND DOCUMENT TYPES: (Article) |
| DocType=All document types; Language=All languages; |
| #3 ((TS=(health-related quality of life))) AND DOCUMENT TYPES: (Article) |
| DocType=All document types; Language=All languages; |
| #2 ((TS=QOL)) AND DOCUMENT TYPES: (Article) |
| DocType=All document types; Language=All languages; |
| #1 (TS=(quality of life)) AND DOCUMENT TYPES: (Article) |
| DocType=All document types; Language=All languages; |
