# Supplementary material for: Informing the measurement of wellbeing among young people living with HIV in sub-Saharan Africa for policy evaluations: a mixed-methods systematic review
Source: Health Qual Life Outcomes. 2020 May 5;18:120. doi: 10.1186/s12955-020-01352-w (PMC7201613; doi:10.1186/s12955-020-01352-w)
Supplement: Supplementary file 8 — Additional file 8. Search strategy- ProQuest. [file 12955_2020_1352_MOESM8_ESM.docx]

Additional file 8: Search strategy (PROQUEST- International Bibliography of the Social Sciences)

| ((((((ab(wellbeing) OR ab(well-being) OR ab(happiness) OR ab("life satisfaction") OR ab("quality of life") OR ab ("psychological functioning") OR ab("lived experience") AND ti(HIV OR HIV/AIDS OR HIV-POSITIVE OR HIV-INFECTED)) AND stype.exact("Scholarly Journals")) AND peer(yes)) AND stype.exact("Scholarly Journals")) AND ab("Angola" OR "Benin" OR "Botswana" OR "Burkina Faso" OR "Burundi" OR "Cameroon" OR "Cape Verde" OR "Central African Republic" OR "CHAD" OR "Comoros" OR "Congo" OR "Congo Democratic Republic" OR "Djibouti" OR "Equatorial Guinea" OR "Eritrea" OR "Ethiopia" OR "Gabon" OR "Gambia" OR "Ghana" OR "Guinea" OR "Guinea-Bissau" OR "Cote d'Ivoire" OR "Ivory Coast" OR "Kenya" OR "Lesotho" OR "Liberia" OR "Madagascar" OR "Malawi" OR "Mali" OR "Mozambique" OR "Namibia" OR "Niger" OR "Nigeria" OR "sago tome and Principe" OR "Rwanda" OR "Senegal" OR "Seychelles" OR "Sierra Leone" OR "Somalia" OR "South Africa" OR "South Sudan" OR "Sudan" OR "Swaziland" OR "Tanzania" OR "Togo" OR "Uganda" OR "Zambia" OR "Zimbabwe" OR "sub-Saharan Africa")) AND peer(yes)) AND (rtype.exact("Journal Article") AND PEER(yes)) |
| --- |
