# Supplementary material for: Informing the measurement of wellbeing among young people living with HIV in sub-Saharan Africa for policy evaluations: a mixed-methods systematic review
Source: Health Qual Life Outcomes. 2020 May 5;18:120. doi: 10.1186/s12955-020-01352-w (PMC7201613; doi:10.1186/s12955-020-01352-w)
Supplement: Supplementary file 9 — Additional file 9. Search strategy- IAS conference abstract archive. [file 12955_2020_1352_MOESM9_ESM.docx]

Additional file 9- Search strategy-IAS Conference abstracts

http://www.abstract-archive.org/

| **Search number** |  |
| --- | --- |
|  | wellbeing or well being |
|  | *Filter-Title, contains wellbeing or well being* |
|  | quality of life or qol |
|  | Filter-Title, contains quality of life or qol |
|  | Lived experiences |
