# Supplementary material for: Informing the measurement of wellbeing among young people living with HIV in sub-Saharan Africa for policy evaluations: a mixed-methods systematic review
Source: Health Qual Life Outcomes. 2020 May 5;18:120. doi: 10.1186/s12955-020-01352-w (PMC7201613; doi:10.1186/s12955-020-01352-w)
Supplement: Supplementary file 10 — Additional file 10. Search strategy- Other databases. [file 12955_2020_1352_MOESM10_ESM.docx]

Additional file 10- Search strategy (Other)

| **Database** | **Type** | **Link** | **Search strategy** |
| --- | --- | --- | --- |
| **Dissertations and Theses A&I** | dissertations | https://www.proquest.com/libraries/academic/dissertations-theses/ | Wellbeing AND QOL AND Mental Health  AND HIV  Filter- SSA countries |
|  |  |  |  |
| **World Cat** | dissertations | https://www.worldcat.org/search | Happiness and HIV  Life satisfaction and HIV  (“Quality of life”) and HIV and Africa  Filter- year (2000-2019) |
|  |  |  |  |
| **OECD** | working papers | http://www.oecd.org | Wellbeing AND HIV  Filter- Health, 2000-2019, journals and working papers |
|  |  |  |  |
| **NBER** | working papers | https://admin.nber.org | “wellbeing” AND “HIV”  Filter- working papers, any date |
|  |  |  |  |
| **IDEAS** | working papers | https://ideas.repec.org | Wellbeing and HIV  Filter- year (2000-2019) |
|  |  |  |  |
| **Google scholar** | publications | https://scholar.google.co.za/ | “Wellbeing” AND “Adolescents living with HIV”  Filter- year (2000-2019) |
|  |  |  | “Mental Health” AND “Adolescents living with HIV”  Filter- year (2000-2019) |
