# Supplementary material for: Informing the measurement of wellbeing among young people living with HIV in sub-Saharan Africa for policy evaluations: a mixed-methods systematic review
Source: Health Qual Life Outcomes. 2020 May 5;18:120. doi: 10.1186/s12955-020-01352-w (PMC7201613; doi:10.1186/s12955-020-01352-w)
Supplement: Supplementary file 11 — Additional file 11. Table 4. Quantitative review outcomes- additional. [file 12955_2020_1352_MOESM11_ESM.docx]

| **Additional file 11: Results from studies included in the quantitative synthesis (N=10)- correlates associated with wellbeing or mental health among YPLHIV in SSA using second scale** | | | | |
| --- | --- | --- | --- | --- |
| **Author year** | **Regression technique** | **Outcome 2 (scale)** | **Univariable/Bivariable analysis**  **(effect size, 95% CI, p value)^$^** | **Multivariable analysis**  **(effect size, 95% CI, p values) ^$^** |
| (Abebe et al., 2019) | Logistic regression | Nil |  |  |
| (Dow et al., 2016) | Negative binomial regression | Total difficulties-(SDQ) | - Not in school   (MR: 1.23, 95% CI 1.02–1.46; p = .03)   - Stigma (per 1 point)   (MR: 1.03, 95% CI 1.02–1.05; p < .001)   - Incomplete adherence   (MR: 1.34, 95% CI 1.11–1.62; p = .002) | - Stigma (per 1 point)   (MR: 1.03, 95% CI 1.01–1.05; p < .001   - Incomplete adherence   (MR: 1.38, 95% CI 1.15–1.65; p = .001) |
| (Earnshaw et al., 2018) | Poisson regression | Nil |  |  |
| (Gaitho et al., 2018) | Linear regression | Nil |  |  |
| (Gentz et al., 2017) | Hierarchical multiple linear regression | Sub-domains-(SDQ) | *Emotional problems*   - Female - (β= 0.255 , 95% CI NR, p<0.05) | *Peer problems-Final model*   - Social support (β= -0.348, 95% CI NR, p<0.001)   *Conduct problems- Final model*   - Child assets   (β= -0.301, 95% CI NR, p<0.01)  *Emotional problems-Final model*   - HIV-stigma - (β= 0.308, 95% CI NR, p<0.01) |
| (Kim et al., 2015) | Linear/  logistic regression | Depressive symptoms (CDSR-R) |  | Final model   - Older age   (OR: 1.23, 95% CI 1.07-1.42, p = 0.004)   - Not in school/junior primary   (OR: 3.30, 95% CI 1.54-7.05, p = 0.005)   - Bullied for taking medication - (OR: 4.20, 95% CI 2.29-7.69, p<0.0001) |
| (Mbalinda et al., 2015) | Logistic regression | Mental health functioning-(MOS-HIV) | NR | - Primary   (aOR: 3.3, 95% CI 1.18-9.38, p =0.02)   - Secondary   (aOR: 5.3, 95% CI 1.86-15.41, p = <0.00)   - Northern region   (aOR:0.50, 95% CI 0.32-0.78; p = <0.001)   - Currently on ARVs   (aOR: 3.9, 95% CI 2.22-6.92; p < 0.001)   - Wants to have a child in the future   (aOR: 1.7, 95% CI 1.05-3.00; p=0.03)   - Parent and adolescent not communicating on sexuality issues   (aOR: 0.6, 95% CI 0.40-0.89; p=0.01   - Has a friend who is smoking cigarette   (aOR: 0.57, 95% CI 0.34-0.98; p =0.04)   - Dissatisfied with the sexual reproductive health service   (aOR: 0.34, 95% CI 0.18-0.62; p<0.00) |
| (Mutumba et al., 2017) | Hierarchical multiple linear regression | Nil |  |  |
| (Okawa et al., 2018) | Logistic regression (multiple) | Nil |  |  |
| (Woollett et al., 2017) | No formal regression, calculated relative risks using Altman's formula | Anxiety symptoms (RCMAS-2) | - Been hit   (RR: 2.00, 95% CI NR, p 0.00)   - Been inappropriately touched   (RR: 1.77, 95% CI NR; p < .001)   - Do not feel like they control their future   (RR: 2.75, 95% CI NR; p < .001)   - Do not feel safe at home   (RR: 2.92, 95% CI NR; p < .001)   - Do not have a dream   (RR: 2.25, 95% CI NR; p < .001)   - Do not have a safe place in the community for adolescents   (RR: 2.02, 95% CI NR; p < .001)   - Experienced forced sex (RR:3.01, 95% CI NR; p =0.01) - Experienced peer violence outside of school (RR:1.62, 95% CI NR; p =0.02) - Reports any form of suicidality   (RR: 2.35, 95% CI NR; p < .001)   - Think about a way to kill themselves   (RR: 2.23, 95% CI NR; p < .001)   - Think about killing themselves   (RR: 1.95, 95% CI NR; p < .001)   - Try to kill themselves   (RR: 2.52, 95% CI NR; p < .001)   - Want to hurt themselves   (RR: 1.97, 95% CI NR; p < .001)   - Wish they were dead - (RR: 2.54, 95% CI NR; p < .001 =1) | - NR |

NR=Not reported, MR=Mean ratio, OR=Odds ratio, aOR= adjusted odds ratio, CI=Confidence interval, QoL=quality of life, RR= risk ratio, $= factors considered statistically significant (as per the study’s definition) are only presented, *= interaction terms
